# Regulation of ncRNAs involved with ferroptosis in various cancers

**DOI:** 10.3389/fgene.2023.1136240

**Published:** 2023-03-29

**Authors:** Chenxi Hu, Xiangbo Zeng, Yuanchao Zhu, Zehai Huang, Jiacheng Liu, Ding Ji, Zaosong Zheng, Qiong Wang, Wanlong Tan

**Affiliations:** ^1^ Department of Urology, Nanfang Hospital, Southern Medical University, Guangzhou, Guangdong, China; ^2^ Department of Infectious Diseases, Peking University Hepatology Institute, Peking University People’s Hospital, Beijing, China; ^3^ Department of Otolaryngology, The First Affiliated Hospital, Sun Yat-sen University, Guangzhou, China

**Keywords:** ferroptosis, circular RNA, microRNA, lnc RNA, chimeric RNA

## Abstract

As a special pattern of programmed cell death, ferroptosis is reported to participate in several processes of tumor progression, including regulating proliferation, suppressing apoptotic pathways, increasing metastasis, and acquiring drug resistance. The marked features of ferroptosis are an abnormal intracellular iron metabolism and lipid peroxidation that are pluralistically modulated by ferroptosis-related molecules and signals, such as iron metabolism, lipid peroxidation, system Xc^−^, GPX4, ROS production, and Nrf2 signals. Non-coding RNAs (ncRNAs) are a type of functional RNA molecules that are not translated into a protein. Increasing studies demonstrate that ncRNAs have a diversity of regulatory roles in ferroptosis, thus influencing the progression of cancers. In this study, we review the fundamental mechanisms and regulation network of ncRNAs on ferroptosis in various tumors, aiming to provide a systematic understanding of recently emerging non-coding RNAs and ferroptosis.

## 1 Introduction

Cell death has been reported to be closely related to cell growth and development, tissue repair, and various physiological processes (Fuchs and Steller, 2011). Cell death includes two significant forms: accidental cell death and regulated cell death (Galluzzi et al., 2018). Accidental cell death is triggered by intensively destructive physical and chemical factors. Correspondingly, regulated cell death could be controlled by external intervention and internal gene changes. There are two regulated cell death forms: apoptotic and non-apoptotic forms. Ferroptosis was first reported as a specific erastin-triggering and iron-dependent non-apoptotic cell death in 2012 (Dixon et al., 2012). Increasing studies have shown that ferroptosis is a pattern of programmed cell death (PCD) characterized by abnormal intracellular iron metabolism and lipid destruction of the cell membrane, which is entirely different from apoptosis or necroptosis (Yang and Stockwell, 2016).

Additionally, ferroptosis could be induced by some small molecules, such as erastin and RAS-selective lethal (RSL) (Dixon et al., 2012). The accumulation of intracellular iron ions is another trigger for ferroptosis. Ferroptosis could be inhibited by antioxidant factors. System Xc^−^ protects the cell from ferroptosis *via* importing cystine and increasing the biosynthesis of GSH (Lewerenz et al., 2013). GPX4 inhibits ferroptosis by decreasing phospholipid hydroperoxide and repressing lipoxygenase-mediated lipid peroxidation (Yang et al., 2014). Nrf2 can repress ferroptosis by indirectly regulating lipid oxidation (Sun et al., 2016). Reactive oxygen species (ROS) can lead to lipid peroxidation and, hence, destroy the cell membrane, leading to ferroptosis (de Carvalho and Caramujo, 2018). Ferroptosis is involved with the occurrence and progression of many diseases, such as neurodegenerative diseases (Belaidi and Bush, 2016), ischemia (Guan et al., 2019), and cancers (Xi et al., 2022; ZhangLncRNA et al., 2022). In recent years, a large number of studies have shown that non-coding RNAs (ncRNAs) could affect tumor progression by regulating ferroptosis. ncRNA is a functional RNA molecule that is not translated into a protein. A growing number of studies have developed and confirmed that ncRNAs are an important regulator of ferroptosis. Here, we reviewed the fundamental mechanisms and regulation networks of ncRNAs on ferroptosis, aiming to provide a systematic understanding of the recently emerging non-coding RNAs and ferroptosis in various tumors (Figure 2; Table 1).

**TABLE 1:** Regulatory pathways and functions of ferroptosis-related ncRNAs in various cancers.

Cancer types	ncRNAs	Molecular mechanism	Biological function	References
Lung cancer	miR-27a-3p	Inhibit SLC7A11	Induces ferroptosis	Lu et al. (2021b)
	miR-4443	METTL3/FSP1	Inhibits ferroptosis and induces cisplatin resistance	Song et al. (2021)
	miR-302a-3p	Target ferroportin	Induces ferroptosis and inhibits NSCLC cell growth and proliferation	Wei et al. (2021)
	miR-6077	Keap1-Nrf2-SLC7A11/NQO1	Inhibits ferroptosis and induces CDDP/PEM resistance	Bi et al. (2022)
	lncRNA H19	miR-19b-3p/FTH1	Inhibits ferroptosis	Zhang et al. (2022a)
	lncRNA T-UCR Uc.339	miR-339/SLC7A11	Inhibits ferroptosis and promotes metastasis	ZhangLncRNA et al. (2022)
	lncRNA GSEC	miRNA-101-3p/CISD1	Promotes LUAD cell growth and migration	Jiang et al. (2021)
	circDTL	miR-1287-5p/GPX4	Inhibits ferroptosis and promotes NSCLC progress	Shanshan et al. (2021)
Esophageal cancer	lncRNA BBOX1-AS1	miR-513a-3p/SLC7A11	Inhibits ferroptosis and promotes ESCC cell proliferation and invasion	Pan et al. (2022)
	circPVT1	miR-30a-5p/FZD3	Inhibits ferroptosis and 5-FU chemosensitivity	Yao et al. (2021a)
	circBCAR3	miR-27a-3p/TNPO1	Promotes ferroptosis	Xi et al. (2022)
Gastric cancer	miR-375	Target SLC7A11	Induces ferroptosis and inhibits stemness of GC cells	Ni et al. (2021)
	exo-miR-522	Decrease ALOX15	Inhibits ferroptosis and promotes cisplatin resistance	Zhang et al. (2020b)
	miR-4715-3p	AURKA/GPX4	Promotes ferroptosis, UGC cell death, and cisplatin sensitivity	Gomaa et al. (2019)
	lncRNA PMAN	Stabilize SLC7A11	Inhibits ferroptosis and promotes tumor development	Lin et al. (2022)
	lncRNA CBSLR	CBS/ACSL4	Inhibits ferroptosis	Yang et al. (2022)
	lncRNA BDNF-AS	WDR5/FBXW7/VDAC3	Inhibits ferroptosis and promotes GC formation and PM	Huang et al. (2022)
Colorectal cancer	miR-545	Decrease transferrin	Inhibits ferroptosis and promotes CRC development	Zheng et al. (2021)
	miR-15a-3p	Target GPX4	Promotes ferroptosis	Liu et al. (2022a)
	miR-19a	Target IREB2	Inhibits ferroptosis and promote CRC development	Fan et al. (2022)
	LINC00239	Keap1/Nrf2	Inhibits ferroptosis and promotes CRC cell growth and proliferation	Han et al. (2022)
	LINC01606	SCD1-Wnt/β‐catenin-TFE3 loop	Inhibits ferroptosis and promotes colon cancer development	Luo et al. (2022)
	circ0007142	miR-874-3p/GDPD5	Inhibits ferroptosis and promotes cell proliferation	Wang et al. (2021a)
Pancreatic cancer	lncRNA A2M-AS1	Interact with PCBP3	Promotes ferroptosis and inhibits pancreatic cancer development	Qiu et al. (2022)
HCC	miR-23a-3p	ETS1/miR-23a-3p/ACSL4	Inhibits ferroptosis and promotes sorafenib resistance	Lu et al. (2022)
	miR-214-3p	Target ATF4	Promotes ferroptosis	Bai et al. (2020)
	lncRNA HEPFAL	Decrease SLC7A11	Promotes ferroptosis and inhibits tumor proliferation and migration	Zhang et al. (2022b)
	lncRNA GABPB1-AS1	Interact with GABPB1	Promotes ferroptosis and HepG2 cell death	Qi et al. (2019)
	lncRNA NEAT1	miR-362-3p/MIOX	Promotes ferroptosis and HCC development	Zhang et al. (2022c)
	lncRNA HULC	miR-3200-5p/ATF4	Inhibits ferroptosis	Guan et al. (2022)
	LINC01134	Nrf2/GPX4	Inhibits ferroptosis and oxaliplatin resistance	Kang et al. (2022)
	circcIARS	Interact with ALKBH5	Promotes ferroptosis	Liu et al. (2020)
	circIL4R	miR-541-3p/GPX4	Inhibits ferroptosis and promotes tumorigenesis	Xu et al. (2020)
	circ0097009	miR-1261/SLC7A11	Inhibits ferroptosis and promotes HCC development	Lyu et al. (2021)
RCC	miR-4735-3p	Targeting SLC40A1	Promotes ferroptosis	Zhu et al. (2022)
	miR-324-3p	Reduce GPX4	Induces ferroptosis of RCC cells	Yu et al. (2022)
Bladder cancer	lncRNA RP11-89	miR-129-5p/PROM2	Inhibits ferroptosis and promote tumorigenesis	Luo et al. (2021)
	circST6GALNAC6	HSPB1/p38 MAPK	Promotes ferroptosis and inhibits tumor development	Wang et al. (2022a)
Prostate cancer	miR-15a	Downregulate GPX4	Promotes ferroptosis and inhibits prostate cancer cell proliferation	Xu et al. (2022a)
	lncRNA PCAT1	c-Myc/miR-25-3p/SLC7A11	Inhibits ferroptosis and promotes docetaxel resistance	Jiang et al. (2022a)
	lncRNA OIP5-AS1	miR-128-3p/SLC7A11	Inhibits ferroptosis and promotes cell growth	Zhang et al. (2021c)
Breast cancer	miR-5096	Target SLC7A11	Promotes ferroptosis and inhibits cancer development	Yadav et al. (2021)
	miR-324-3p	Decrease GPX4	Promotes ferroptosis	Hou et al. (2021)
	circRHOT1	miR-106a-5p/STAT3	Inhibits ferroptosis and promotes malignant development	Zhang et al. (2021b)
	circGFRA1	miR1228/AIFM2	Inhibits ferroptosis and promotes cancer development	Bazhabayi et al. (2021)
Ovarian cancer	miR-424-5p	Target ACSL4	Inhibits ferroptosis	Ma et al. (2021b)
	lncRNA ADAMTS9-AS1	miR-587/SLC7A11	Inhibits ferroptosis and promotes epithelial ovarian cancer development	Cai et al. (2022)
Cervical Cancer	circLMO1	miR-4291/ACSL4	Promote ferroptosis and inhibits cell proliferation and invasion	Ou et al. (2022)
	circEPSTI1	miR-375/409-3p/515-5p-SLC7A11	Inhibits ferroptosis and promotes cell proliferation	Wu et al. (2021b)
	circACAP2	miR-193a-5p/GPX4	Inhibits ferroptosis and promotes cell proliferation	Liu et al. (2022b)
AML	LINC00618	Target SLC7A11	Promotes ferroptosis and inhibits AML development	Wang et al. (2021c)
	circKDM4C	hsa-let-7b-5p/p53	Promotes ferroptosis and inhibits AML development	Dong et al. (2021)
Glioblastoma	miR-147a	Target SLC40A1	Induces ferroptosis	Xu et al. (2022b)
	miR-670-3p	Target ACSL4	Inhibits ferroptosis	Bao et al. (2021)
	lncRNA TMEM161B-AS1	Hsa-miR-27a-3p/FANCD2, CD44	Inhibits ferroptosis and promotes GBM development	Chen et al. (2021b)
	circLRFN5	PRRX2/GCH1	Promotes ferroptosis and inhibits GBM development	Jiang et al. (2022b)
Melanoma	miR-9	Downregulate GOT1	Inhibits ferroptosis	Zhang et al. (2018b)
	miR-137	Target SLC1A5	Inhibits ferroptosis	Luo et al. (2018)
	miR-130b-3p	DKK1-dependent Nrf2/HO-1	Inhibits ferroptosis	Liao et al. (2021)
	miR-21-3p	Target TXNRD1	Induces ferroptosis and increased anti-PD-1 immunotherapy sensitivity	Guo et al. (2022)

### 1.1 Major mechanisms in ferroptosis

#### 1.1.1 Iron metabolism

The accumulation of iron could cause excess ROS production *via* the Fenton reaction, inducing ferroptosis (Dixon and Stockwell, 2014). Therefore, homeostasis of intracellular iron is a key factor affecting ferroptosis. Intracellular iron homeostasis is regulated finely and dynamically by iron regulatory proteins (IRPs), which depend on their ability to bind to iron-responsive elements (IREs) (Zhang et al., 2014). In general, transferrin binds to extracellular iron to transfer iron from extracellular to intracellular in a transferrin receptor-dependent manner (Gao et al., 2015). Ferritin degradation is another alternative method to promote the concentration of intracellular iron (Hou et al., 2016). Correspondingly, ferritin-containing multivesicular bodies (MVBs) and exosomes are the main vehicles for transporting intracellular iron to the extracellular space (Brown et al., 2019). Solute carrier family 40 member 1 (SLC40A1), an iron transporter, has a significant effect on iron export (Donovan et al., 2005), which could be regulated by transcription factor BTB and CNC homology 1 (BACH1) (Nishizawa et al., 2020). Other studies also reported that Tau protein and ferritin chain proteins, FTH1 and FTL1, participate in the iron efflux adjustment (Tuo et al., 2017; Nishizawa et al., 2020), but the specific underlying mechanism needs to be further explored. NCOA4 is a ferritin cargo receptor, and NCOA4-mediated ferritinophagy is required to maintain intracellular and systemic iron homeostasis and, thus, iron-dependent physiological processes (Gao et al., 2016; Santana-Codina et al., 2021). In summary, intracellular iron homeostasis is a process involving multiple pathways, which coordinate and regulate intracellular iron levels, thereby affecting the level of ferroptosis.

#### 1.1.2 Lipid peroxidation

Lipids participate in the formation of biomembranes and play an important role in molecular signal transmission. Ferryl radical, peroxynitrite (ONOO^−^), and many instable molecules that require hydrogen atoms can combine with allylic hydrogen atoms in polyunsaturated fatty acids (PUFAs), thus forming lipid radicals (Higdon et al., 2012). Subsequently, lipid alkoxyl (LO•) and lipid peroxyl (LOO•) radicals can combine with other PUFAs, leading to a continuously expanding reaction (Porter et al., 1995). The accumulation of lipid peroxidation products and the relative inadequacy of GPX4/GSH cause ferroptosis (Kagan et al., 2017). Recent studies suggest that PUFAs, including arachidonoyl (AA) and adrenoyl (AdA), are sensitive to oxidation and participate in the ferroptosis process (Yang and Stockwell, 2016; Kagan et al., 2017). Kagan, V. E. et al. discovered oxygenated di-acyl AA/AdA-containing phosphatidylethanolamines (PEs) acted as pivotal ferroptosis signals *via* LC-MS/MS identification (Kagan et al., 2017). Acyl-CoA synthetase long-chain family member 4 (ACSL4) can catalyze AA/AdA into AA/AdA-CoA (Küch et al., 2014; Kagan et al., 2017). Then, lyso-phosphatidylcholine acyltransferase 3 (LPCAT3) esterifies AA/AdA-CoA to PE (Yang et al., 2016; Kagan et al., 2017). Interestingly, although AA and AdA can be oxygenated by LOX, as well as cyclooxygenases (COX) and cytochrome P450 (CYP450), ferroptosis is inhibited only when LOX is suppressed (Kagan et al., 2017), suggesting that LOX plays a distinctive role in ferroptosis. Therefore, lipid metabolism plays an important role in ferroptosis, and lipid peroxidation products participate in ferroptosis directly.

#### 1.1.3 System Xc^-^

System Xc^−^ consists of solute carrier family 7 member 11 (SLC7A11 or xCT, catalytic subunit) and solute carrier family 3 member 2 (SLC3A2, regulatory subunit) and possesses high selectivity for cystine and glutamate transport, especially for the anionic form. System Xc^−^ can absorb one cystine and export one glutamate simultaneously (Sato et al., 1999). Cystine can be catalyzed into cysteine. Cysteine, glutamic acid, and glycine can be synthetized into GSH, which prevents the cell membrane or biomacromolecules from peroxide destruction or ferroptosis (Lewerenz et al., 2013). However, the small-molecule erastin and anti-cancer drug sorafenib can inhibit system Xc^−^ to induce ferroptosis. Specifically, erastin causes the depletion of intracellular cystine to repress GSH synthesis. In addition, erastin also initiates endoplasmic reticulum (ER) stress (Dixon et al., 2014). The expression of system Xc^−^ can be regulated at the transcription or translation level. Chen, D. et al. uncovered that transcription factor 4 (ATF4) increases system Xc^−^ expression and glutamate exportation to inhibit ferroptosis (Chen et al., 2017a). Nrf2 signals also participate in system Xc^−^ regulation, which acts as a transcription factor of system Xc^−^. Nrf2 overexpression or Keapl knockdown can upregulate system Xc^−^. Nrf2 can repress ferroptosis by facilitating glutamate exportation (Fan et al., 2017). In Zhang, Y. et al.’s research, SLC7A11 was a significant downstream target of tumor suppressor BRCA1-associated protein 1 (BAP1). BAP1 can encode a nuclear deubiquitinating (DUB) enzyme that participates in the composition of the polycomb repressive deubiquitinase (PR-DUB) complex. BAP1 promotes lipid peroxidation *via* repressing SLC7A11 expression in a PR-DUB complex-repressing histone 2A ubiquitination (H2Aub) manner (Zhang et al., 2018a). Moreover, epigenetic modification involving with system Xc^−^ was also reported. N^6^-methyladenosine (m^6^A) “reader” YT521-B homology containing 2 (YTHDC2) repressed the system Xc^−^ regulatory subunit SLC3A2 *via* decreasing the stability of homeobox A13 (HOXA13), which elevated SLC3A2 expression (Ma et al., 2021a). In summary, system Xc^−^ inhibits the ferroptosis process, and the subunit SLC7A11 is the principal and common regulator used to investigate ferroptosis.

#### 1.1.4 GPX4

**FIGURE 1:**
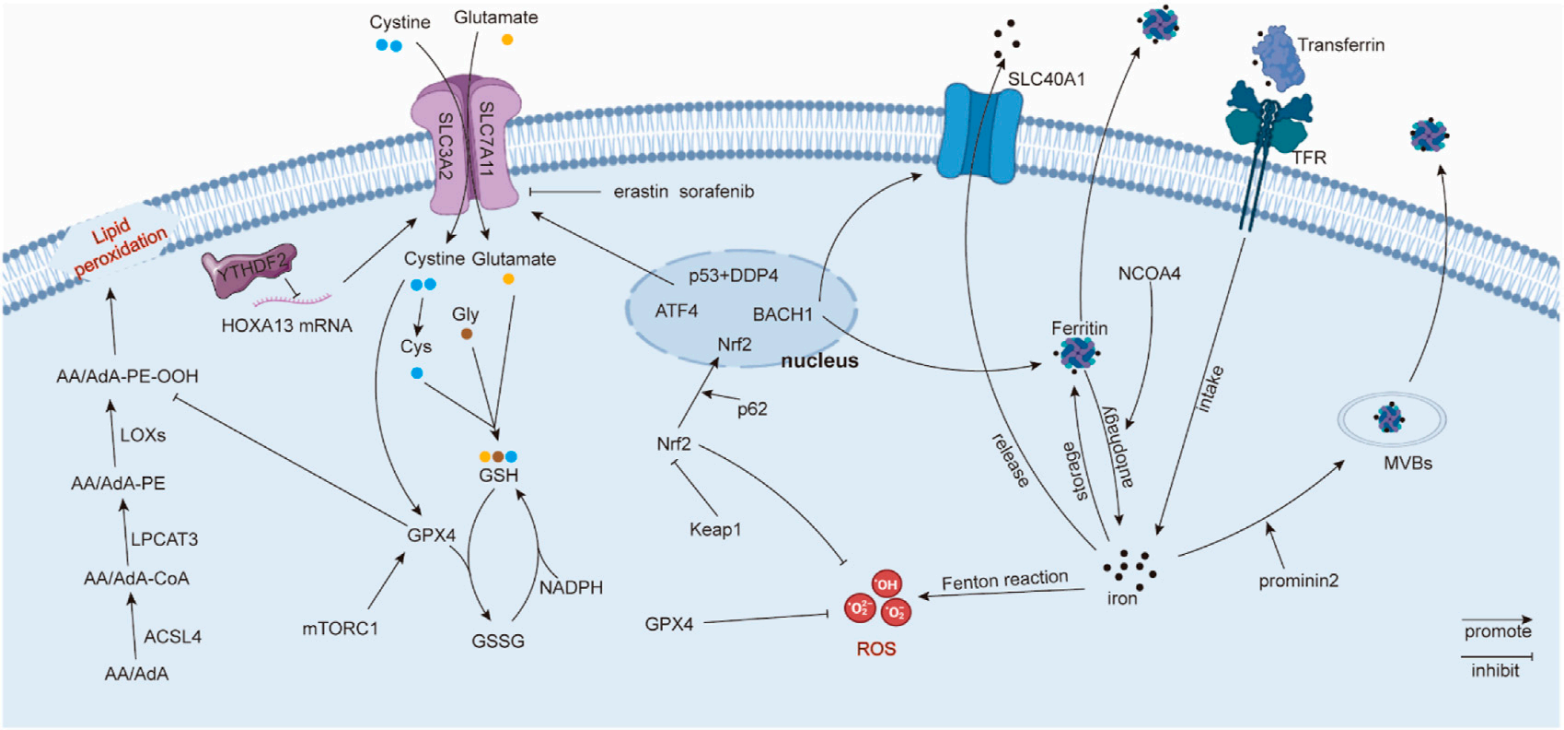
Regulatory mechanisms in ferroptosis. Solute carrier family 40 member 1 (SLC40A1); ferritin heavy chain 1 (FTH1); ferritin light chain 1 (FTL1); reactive oxygen species (ROS); phosphatidylethanolamines (PE); arachidonoyl (AA) adrenoyl (AdA); lipoxygenases (LOXs); polyunsaturated fatty acids (PUFA); phospholipids (PL-OOH); and lyso-phosphatidylcholine acyltransferase 3 (LPCAT3).

GPX4 belongs to selenium-dependent glutathione peroxidases, and it can catalyze phospholipid and cholesterol hydroperoxides to appropriate alcohols relying on its selenocysteine residue and GSH (Maiorino et al., 2018). GPX4 catalyzes GSH to oxidized glutathione (GSSG). Correspondingly, glutathione reductase and NADPH/H+ work together to catalyze GSSG back to GSH, and this provides an effective cycle for GSH metabolism (Circu and Aw, 2008). GPX4 can prevent cell membranes from lipid peroxidation, while small ferroptotic inducers such as erastin, RSL3, and l-buthionine sulfoximine (BSO) can inhibit this process (Yang et al., 2014). Other studies found that GPX4 helps to decrease the production of PUFA hydroperoxides and phospholipids (PL-OOH) (Imai and Nakagawa, 2003), as well as inhibit the accumulation of oxygenated PE (Kagan et al., 2017). Additionally, some molecular signals participate in GPX4 regulation. Zhang, Y. et al. found that system Xc^−^ dependent on cystine can increase GPX4 synthesis through the Rag-mTORC1-4EBP pathway, thus inhibiting ferroptosis (Zhang et al., 2021a). The ferroptosis inducer Fin56 can inhibit the translation of GPX4, thus inducing ferroptosis (Sun et al., 2021), while KLF2 increases the transcription of GPX4 to repress ferroptosis (Lu et al., 2021a). Moreover, the transcription factor Nrf2 can be recruited to the GPX4 promoter to elevate its expression (Kang et al., 2022). In brief, GPX4 can prevent cancer cells from ferroptosis by countering lipid peroxidation. The blocking of GPX4 enhances cell ferroptosis, while the overexpression of GPX4 reverses this impact (Yang et al., 2014) (Figure 1).

#### 1.1.5 Others

ROS accumulation leads to lipid peroxidation, thus promoting cell membrane destruction and ferroptosis (Dixon et al., 2012). ROS can be generated from oxygen metabolism, iron-mediated Fenton reaction, or lipid peroxidation, including superoxide anions (O_2_^•−^), hydroxyl radicals (•OH), NO, and RO^•^ (Dixon et al., 2012; Brandes et al., 2014). ROS is also reported to be derived from some enzymatic reactions mediated by oxidases, such as cyclooxygenases (COXs), lipoxygenases (LOXs), and cytochrome P450 (Florean et al., 2019). Erastin and RSL can accumulate lipid ROS, thus inducing ferroptosis (Dixon et al., 2012).

Kelch-like ECH-associated protein 1 (Keap1) can bind to both Nrf2 and the actin cytoskeleton to retain Nrf2 in the cytoplasm (Wakabayashi et al., 2003), and Nrf2 is an indispensable regulator of reduction–oxidation balance. The Cullin-3 E3 ligase complex is a potential upstream of Keap1, which can polyubiquitinate Nrf2 in a Keap1-dependent manner (Kobayashi et al., 2004). Another study uncovered that the tumor suppressor ARF can suppress Nrf2 and its downstream target SLC7A11 to facilitate ferroptosis (Chen et al., 2017b). Moreover, Sun, X. et al. found that p62 can attenuate Nrf2 degradation and accumulate Nrf2 in the nucleus *via* mediating Keap1 inactivation, thus inhibiting ferroptosis (Sun et al., 2016). Moreover, Nrf2 can inhibit ferroptosis *via* increasing iron storage protein ferritin or heme oxygenase 1 (Kerins and Ooi, 2018). Nrf2 can also activate GPX4 (Kang et al., 2022). Therefore, the Keap1/Nrf2 axis acts as a suppressor regulator in ferroptosis.

P53, known as a tumor suppressor, is also associated with ferroptosis. Interestingly, the role of p53 on ferroptosis is still controversial. Jiang, L. et al. reported that p53 can increase susceptibility to ferroptosis by inhibiting SLC7A11 expression and abating cystine uptake (Jiang et al., 2015). On the contrary, Xie, Y. et al. found that p53 represses ferroptosis in human colorectal cancer (CRC) cells. Downregulated p53 can reduce dipeptidyl peptidase 4 (DPP4) in the nucleus, thus indirectly promoting DPP4-mediated lipid peroxidation in plasma and membrane (Xie et al., 2017). Thus, p53 is a complicated factor in ferroptosis, and its distinct regulatory roles in ferroptosis need to be clarified.

### 1.2 ncRNAs in ferroptosis

Different from messager RNA, non-coding RNAs (ncRNAs) cannot be directly translated into bioactive proteins but can regulate the transcription and translation of target transcripts. ncRNAs include microRNAs (miRNAs), long non-coding RNAs (lncRNAs), circular RNAs (circRNAs), Piwi-interacting RNA (piRNA), small nucleolar RNA (snoRNA), and chimeric RNA, but different ncRNAs have distinguishing regulatory mechanisms in ferroptosis.

MiRNAs are small, single-stranded, non-coding RNA molecules containing 21 to 23 nucleotides. MiRNAs typically have an endogenous suppressor effect on gene expression *via* binding to 3’-untranslated regions (3’UTR). MiR-27a-3p (Lu et al., 2021b), miR-339 (ZhangLncRNA et al., 2022), and miR-375 (Ni et al., 2021) were reported to bind to the 3’UTR of SLC7A11 to decrease its expression. MiR-1287-5p (Shanshan et al., 2021) and miR-15a-3p (Liu et al., 2022a) can target GPX4’s 3’UTR to inhibit its antioxidation function. Similarity, miR-23a-3p (Lu et al., 2022) and miR-424-5p (Ma et al., 2021b) are regulators of ACSL4, which can result in the downregulation of PUFA-CoA. MiR-545 is a regulator of transferrin, which can reduce iron intake (Zheng et al., 2021). Therefore, the role of miRNA in ferroptosis mostly depends on its negative regulation of ferroptosis-related genes.

Long non-coding RNAs (lncRNAs) are a type of RNAs, generally defined as transcripts of more than 200 nucleotides that are not translated into protein. LncRNAs regulate gene expression in a variety of ways at the epigenetic, chromatin remodeling, transcriptional, and translational levels. As for ferroptosis, lncRNAs usually interact with miRNAs in a competitive manner, leading to the inhibition of miRNAs. For instance, lncRNA H19 upregulates FTH1 by interacting with miR-19b-3p (Zhang et al., 2022a). LncRNA T-UCR Uc.339 increased SLC7A11 *via* interacting with miR-339 (ZhangLncRNA et al., 2022), and LINC01606 interacts with miR-423-5p to elevate SCD1 expression (Luo et al., 2022). In addition, lncRNAs were also reported to participate in the modulation of mRNA stability and protein ubiquitination. For example, lncRNA PMAN can stabilize SLC7A11 mRNA to promote its translation (Lin et al., 2022). LncRNA HEPFAL can promote SLC7A11 protein ubiquitination, decreasing the level of SLC7A11 (Zhang et al., 2022b). Chao Mao reported a G3BP1-interacting lncRNA that can promote ferroptosis in cancer *via* the nuclear sequestration of p53 (Mao et al., 2018). In short, lncRNAs regulate ferroptosis in multiple ways.

Circular RNA (or circRNA) is a type of single-stranded RNA that, unlike linear RNA, forms a covalently closed continuous loop without 5’ caps or 3’ tails. Most current studies demonstrate that circRNAs function as a sponge for miRNAs, thus regulating ferroptosis-related genes. For example, circPVT1 sponges miR-30a-5p to target FZD3 (Yao et al., 2021a); circBCAR3 sponges miR-27a-3p to upregulate TNPO1 (Xi et al., 2022); and circ0007142 acts as a sponge for miR-874-3p, thus regulating GDPD5 (Wang et al., 2021a); circRHOT1 functions as a sponge for miR-106a-5p to promote the expression of STAT3 (Zhang et al., 2021b). In addition, circRNA was also reported to bind to protein directly to influence ferroptosis. Zhiqian Liu found that hsa_circ_0008367 physically interacts with RNA-binding protein (RBP) ALKBH5, which is a regulator of ferroptosis (Liu et al., 2020). CircST6GALNAC6 binds to the N-terminus of small heat shock protein 1 (HSPB1) and, thus, blocks the erastin-induced phosphorylation of HSPB1 at the Ser-15 site, a phosphorylation site in the protective response to ferroptosis stress (Wang et al., 2022a). CircEXOC5 has a direct binding relationship with PTBP1 to aggravate ferroptosis (Wang et al., 2022b). Up to now, circRNAs have been reported to regulate ferroptosis in the abovementioned two ways. However, we consider the protein-coding and transcriptional regulation ability of circRNAs to be an option for them to affect ferroptosis.

In addition, epigenetic modification N^6^-methyladenosine (m^6^A) was reported to be involved with ncRNA-mediated ferroptosis. Methyltransferase METTL3 can be regulated by miR-4443, and METTL3 reduced ferroptosis suppressor protein 1 (FSP1) *via* m^6^A modification (Song et al., 2021). lncRNA CBSLR destabilized CBS mRNA dependent on m^6^A “reader” protein YTHDF2; thus, decreasing CBS leads to ACSL4 downregulation (Yang et al., 2022).

PiRNAs can combine with piwi proteins to make up a piRNA/piwi complex, which can cause gene silencing *via* interacting with a target transcript (Liu et al., 2019). For instance, the piR-36712/SEPW1P RNA/miR-7/− 324/P53/P21 axis participates in the regulation of breast cancer cell proliferation and invasion (Tan et al., 2019). Considering that ferroptosis is a p53-mediated activity during tumor suppression (Jiang et al., 2015), we believe that piRNAs are another type of ncRNAs that can regulate ferroptosis. tRNAs, rRNAs, snRNAs, and snoRNAs are also contained in the family of non-coding RNAs. However, studies on the relationship between these ncRNAs and ferroptosis are few, even though they signature as biomarkers for multiple tumors (Zhang et al., 2020a). Our previous study identified many novel chimeric RNAs in prostate cancer, which may function as ncRNAs (Wang et al., 2022c). Although we have not yet studied the relationship between these chimeric RNAs and ferroptosis, their parental genes such as GNPDA1 and EEF2 are significant in the process of ferroptosis (Zhong et al., 2020; Dai et al., 2021), suggesting that chimeric RNAs could be a new repertoire for biomarker and drug-target discovery related to ferroptosis.

In summary, the current research on ferroptosis-related ncRNAs is still mainly focused on miRNAs, lncRNAs, and circRNAs. For other types of ncRNAs, further research is still needed.

### 1.3 Ferroptosis in cancers

As a special pattern of programmed cell death, ferroptosis is inconsistent with the characteristics of the unlimited proliferation of cancer cells, suggesting that ferroptosis is a procedure to inhibit tumor progression. It is reported that the abnormal iron homeostasis is involved in many malignant tumors (El Hout et al., 2018). More and more novel ferroptosis-related small molecules or genes have been elucidated participating in tumor progression. FINO2 is an endoperoxide-containing 1,2-dioxolane that can oxidize Fe(II) and repress GPX4 enzymatic catalytic activity indirectly, which leads to extensive lipid peroxidation, aggravating the ferroptosis process of cancer cells (Gaschler et al., 2018). Non-thermal plasma (NTP) can induce lipid peroxidation occurrence and mitochondrial superoxide generation dependent on catalytic Fe(II), thus killing cancer cells *via* ferroptosis (Sato et al., 2019). Certainly, it is common that ferroptosis regulates cancer cell death *via* traditional ferroptosis-related molecules. Ferroptosis inhibits the proliferation, invasion, and migration of cancer cells *via* SLC7A11 (Zhang et al., 2022b), GPX4 (Liu et al., 2022a), SLC40A1 (Zhu et al., 2022), ACSL4 (Ou et al., 2022), and p53 (Dong et al., 2021). In addition, many experimental compounds targeting system Xc^−^, GPX4, and Nrf2 have been used to induce ferroptosis against cancer development (Shen et al., 2018). Ferroptosis can also increase the anti-PD-1 immunotherapy effect (Guo et al., 2022) and the chemotherapeutic sensitivity, such as cisplatin (Roh et al., 2016) and oxaliplatin (Kang et al., 2022). However, therapeutic strategies targeting ferroptosis-related ncRNAs are still inadequate. Therefore, elucidating the role of diverse ncRNAs in different tumors is imperative for the development of new diagnosis and therapeutic strategies.

## 2 Regulation of ncRNAs related with ferroptosis in cancers

### 2.1 Lung cancer

Lung cancer is by far the leading cause of cancer death, accounting for 11.4% of diagnosed cancers and 18.0% of cancer deaths (Sung et al., 2021). MiR-27a-3p is downregulated in NSCLC cells, which can inhibit the expression of SLC7A11 by binding to its 3’-UTR (Lu et al., 2021b). MiR-4443 is an important suppressor of m^6^A methyltransferase METTL3. METTL3 can regulate ferroptosis-related gene FSP1 *via* m^6^A modification, thus leading to cisplatin resistance (Song et al., 2021). Another example is miR-302a-3p, which can bind to the 3’UTR of ferroportin and inhibit its expression. It decreased ferroportin-overloaded intracellular iron and, finally, resulted in lipid peroxidation and ferroptosis (Wei et al., 2021). Bi, G. et al. reported that the miR-6077-Keap1-Nrf2-SLC7A11/NQO1 axis impedes ferroptosis and induces cisplatin (CDDP)/pemetrexed (PEM) resistance (Bi et al., 2022).

LncRNAs and circRNAs also play an important role in lung cancer progression through regulating ferroptosis. For example, lncRNA H19 can bind to miR-19b-3p in a competitive manner, and then, the ferritin heavy chain 1 (FTH1), a miR-19b-3p endogenous target, was activated to reduce intracellular iron followed by the repression of ferroptosis (Zhang et al., 2022a). LncRNA Uc.339 is overexpressed in lung adenocarcinoma, and it has been reported to bind to pre-miR-339 to prevent the maturity of miR-339, and then, SLC7A11, the downstream of miR-339, is upregulated (ZhangLncRNA et al., 2022).

**FIGURE 2:**
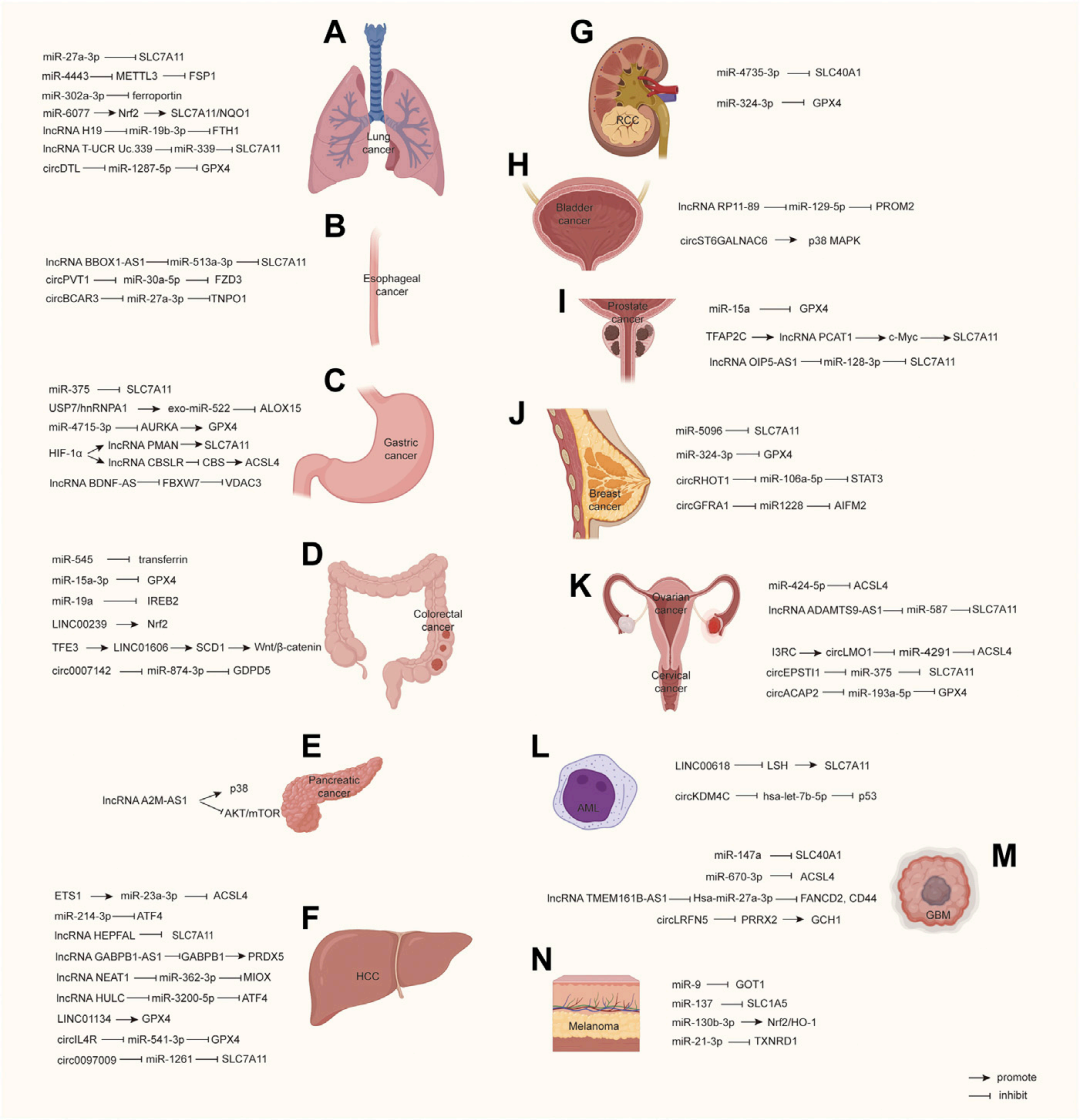
Molecular pathways in ncRNA-regulated ferroptosis among malignant cancers. **(A–N)** propose how ncRNAs regulate ferroptosis-related molecules and signals. HCC, Hepatocellular carcinoma; RCC, Renal cell carcinoma; AML, Acute myeloid leukemia; GBM, Glioblastoma.

In lung adenocarcinoma (LUAD), many bioinformatics analyses predicted a large number of ferroptosis-related lncRNAs related to survival (Yao et al., 2021b; Lu et al., 2021c; Guo et al., 2021). Ferroptosis-related CISD1 plays an oncogene role in promoting LUAD growth and migration, which is regulated by the lncRNA GSEC/miRNA-101-3p/CISD1 axis (Jiang et al., 2021). CircDTL is overexpressed in NSCLC patient tissues and cell lines, and Shanshan, W. et al. found that it can inhibit ferroptosis by targeting the miR-1287-5p/GPX4 axis (Shanshan et al., 2021) (Figure 2A). In conclusion, ncRNA affects the progress of the lung cancer by regulating the expression of ferroptosis-related genes, mainly through the RNA–RNA-binding mechanism. In particular, SLC7A11 is the target of many ncRNAs, suggesting that it may play a more important role in lung cancer compared to other genes.

### 2.2 Esophageal cancer

There were 604,000 new cases of esophageal cancer and 544,000 deaths worldwide in 2020 (Sung et al., 2021). Chunfeng Pan reported that the downregulation of lncRNA BBOX1-AS1 inhibits cell proliferation and metastasis and accelerates cell ferroptosis in esophageal squamous cell cancer (ESCC) by upregulating miR-513a-3p to reduce SLC7A11 expression (Pan et al., 2022). Yao W.’s research suggests a key role for circPVT1 in ESCC 5-FU-chemosensitivity by regulating ferroptosis *via* the miR-30a-5p/FZD3 axis. They found that knocking down circPVT1 increased ferroptosis through downregulating p-β-catenin, GPX4, and SLC7A11, while the inhibition of miR-30a-5p and overexpression of FZD3 reversed the phenotype through their upregulation (Yao et al., 2021a). Yong Xi et al. demonstrated that hypoxia induces the upregulation of E2F7, which transcriptionally activates QKI in esophageal cancer cells. QKI increases the formation of circBCAR3 by juxtaposing the circularized exons. CircBCAR3 binds with miR-27a-3p to promote transportin-1 (TNPO1) expression, thus promoting cancer cell proliferation, migration, and invasion (Xi et al., 2022) (Figure 2B). Up to now, there are few reported ferroptosis-related ncRNAs, suggesting that the role of ncRNA in esophageal cancer may not be as important as other tumors.

### 2.3 Gastric cancer

Gastric cancer caused 769,000 deaths worldwide in 2020, and its incidence rate ranks fifth among cancers in the world (Sung et al., 2021). Ni, H. et al. found that miR-375 can trigger ferroptosis to inhibit GC cell stemness through targeting SLC7A11 (Ni et al., 2021). Haiyang Zhang et al. found that cisplatin and paclitaxel promote miR-522 secretion from CAFs by activating the USP7/hnRNPA1 axis. MiR-522 leads to ALOX15 suppression and decreases ferroptosis in cancer cells, ultimately resulting in decreased chemosensitivity (Zhang et al., 2020b). Gomaa, A. et al. identified a novel epigenetic mechanism mediating the silencing of miR-4715-3p and induction of Aurora kinase A (AURKA) in UGCs. MiR-4715-3p downregulates AURKA by directly targeting its 3’UTR. The inhibition of AURKA or the reconstitution of miR-4715-3p inhibits GPX4 and induces cell ferroptosis (Gomaa et al., 2019).

LncRNAs are also important in ferroptosis in gastric cancer. HIF-1α can bind to the endogenous HRE site in the PMAN promoter, thus upregulating lncRNA PMAN. LncRNA PMAN improves the stability of SLC7A11 mRNA dependent on the cytoplasmic distribution of ELAVL1 (Lin et al., 2022). HIF-1α can also induce the expression of lncRNA CBSLR. CBSLR interacts with YTHDF2 to form a CBSLR/YTHDF2/CBS signaling axis that decreases the stability of CBS mRNA by enhancing the binding of YTHDF2 with the m^6^A-modified coding sequence (CDS) of CBS mRNA. Under decreased CBS levels, the methylation of the ACSL4 protein is reduced, leading to protein polyubiquitination and the degradation of ACSL4, which, in turn, decreases the proferroptosis phosphatidylethanolamine (PE) (Yang et al., 2022). Huang, G. et al. found that lncRNA BDNF-AS is highly expressed in gastric cancer (GC) and peritoneal metastasis (PM) tissues. Their further study demonstrated that BDNF-AS can regulate FBXW7 expression by recruiting WDR5, thus affecting FBXW7 transcription, and FBXW7 regulates the protein expression of VDAC3 through ubiquitination to protect GC cells from ferroptosis (Huang et al., 2022). Moreover, bioinformatics analysis predicted some other ferroptosis-related lncRNAs which can act as prognosis signatures or references for clinical outcomes (Li et al., 2022a; Geng et al., 2022) (Figure 2C). Overall, ferroptosis can be induced when gastric cancer faces environmental stresses, and this effect may be more dependent on ncRNAs. This characteristic is conducive to finding ferroptosis-related ncRNAs in gastric cancer.

### 2.4 Colorectal cancer

More than 1.9 million new CRC cases were diagnosed and the incidence rate of CRC ranked third in 2020 (Sung et al., 2021). Zheng, S. et al. found that miR-545 accelerated CRC cell survival *via* reducing transferrin, and while transferrin overexpression blocked miR-545-induced changes in ROS, MDA, and Fe^2+^ levels in HT-29 and HCT-116 cells, thereby inducing CRC ferroptosis (Zheng et al., 2021). Another study identified that miR-15a-3p promotes ferroptosis *via* directly repressing the expression of GPX4 through binding to the 3’-untranslated region of GPX4, resulting in increased reactive oxygen species levels, intracellular Fe^2+^ levels, and malondialdehyde accumulation *in vitro* and *in vivo* (Liu et al., 2022a). In addition, iron-responsive element-binding protein 2 (IREB2), as an inducer of ferroptosis, is a direct target of miR-19a (Fan et al., 2022).

Many lncRNAs and circRNAs were also reported to influence the progression of CRC through ferroptosis. Han, Y. et al. explored the transcriptomic profiles of lncRNAs in primary CRC tissues and found that LINC00239 is significantly overregulated in colorectal cancer tissues and its overexpression predicts poorer survival and prognosis. Mechanically, LINC00239 plays a novel and indispensable role in ferroptosis by nucleotides 1-315 of LINC00239 to interact with the Kelch domain (Nrf2-binding site) of Keap1, inhibiting Nrf2 ubiquitination and increasing Nrf2 protein stability, thus inhibiting ferroptosis and promoting chemoresistance (Han et al., 2022). Yajun Luo et al. found that LINC01606 protects colon cancer cells from ferroptosis by decreasing the concentration of iron, lipid-reactive oxygen species, and mitochondrial superoxide and increasing the mitochondrial membrane potential. Mechanistically, LINC01606 enhances the expression of stearoyl-CoA desaturase 1 (SCD1), serving as a competing endogenous RNA to modulate miR-423-5p expression, subsequently activating canonical Wnt/β-catenin signaling, and transcription factor binding to IGHM enhancer 3 (TFE3) also increases LINC01606 transcription after recruitment to the promoter regions of LINC01606 (Luo et al., 2022). Wang, Y. et al. uncovered that circ0007142 is a miR-874-3p sponge. MiR-874-3p targets glycerophosphodiester phosphodiesterase domain containing 5 (GDPD5), and the upregulation of GDPD5 reverses the miR-874-3p-triggered tumor inhibition and ferroptosis promotion (Wang et al., 2021a). Bioinformatics analysis also predicted some ferroptosis-related lncRNAs involved with CRC prognosis (Wu et al., 2021a) (Figure 2D). In summary, many ncRNAs are reported to associate to colorectal cancer, and the downstream pathways are also diverse, suggesting that colorectal cancer maybe more sensitive to ferroptosis-related therapies.

### 2.5 Pancreatic cancer

Pancreatic cancer is extremely malignant. There were 496,000 new cases and 466,000 deaths due to its badly poor prognosis (Sung et al., 2021). Xin Qiu et al. found that lncRNA A2M-AS1 can directly interact with poly (rC)-binding protein 3 (PCBP3), which is involved closely with iron metabolism. In addition, the A2M-AS1/PCBP3 axis can facilitate p38 activation and inhibit the phosphorylation of the AKT-mTOR signaling pathway, and the two pathways mentioned above are reported to participate in regulating ferroptosis (Qiu et al., 2022). The linc02432/Hsa-miR-98-5p/HK2 axis is another example that can inhibit ferroptosis and predict immune infiltration, tumor mutation burden, and drug sensitivity in pancreatic adenocarcinoma (Tan et al., 2022). Bioinformatics analysis identified some ferroptosis-related lncRNAs in pancreatic cancer, such as lncZNF236-DT, lncCASC8, and lncPAN3-AS1 (Ping et al., 2022) (Figure 2E). Current studies have not reported on the relationship between ncRNAs and classic ferroptosis genes, and there are still no abundant ncRNAs reported to regulate ferroptosis in pancreatic cancer, which means the effect of ferroptosis therapies may not be inadequate for this type of cancer.

### 2.6 Hepatocellular carcinoma

Liver cancer is the sixth most common cancer worldwide, with approximately 906,000 new cases and 830,000 deaths in 2020 (Sung et al., 2021). Lu, Y. et al. reported that the ETS1/miR-23a-3p/ACSL4 axis contributes to sorafenib resistance in hepatocellular carcinoma (HCC) through regulating ferroptosis. ETS1 activated miR-23a-3p on its promoter transcriptionally by recognizing the DNA-binding GGAA/T sequence (Lu et al., 2022). MiR-214-3p increases intracellular iron accumulation and reduces GSH by inhibiting ATF4, thus facilitating ferroptosis (Bai et al., 2020).

LncRNA HEPFAL is reduced in HCC, and it can promote ferroptosis by reducing the stability of SLC7A11 and increasing the levels of intracellular lipid ROS and iron. In addition, lncRNA HEPFAL increases the sensitivity of erastin-induced ferroptosis, which may be related to mTORC1 (Zhang et al., 2022b). Erastin upregulates the GABPB1 antisense chain lncRNA GABPB1-AS1, which downregulates the GABPB1 protein levels by blocking GABPB1 mRNA recruitment to polysomes and binding with eIF4A, leading to the downregulation of the gene encoding peroxiredoxin-5 (PRDX5) peroxidase and the eventual suppression of the cellular antioxidant capacity (Qi et al., 2019). Erastin and RSL3 increase lncRNA NEAT1 expression by promoting the binding of p53 to the NEAT1 promoter. NEAT1 can competitively interact with miR-362-3p to avoid binding to MIOX’ 3’UTR, elevates MIOX-mediated ROS production, and decreases the intracellular levels of NADPH and GSH, resulting in enhanced ferroptosis (Zhang et al., 2022c). LncRNA HULC was found to function as a ceRNA of miR-3200-5p, and miR-3200-5p regulates ferroptosis by targeting ATF4, resulting in the inhibition of proliferation and metastasis within the HCC cells (Guan et al., 2022). LINC01134 is reported to be positively correlated with GPX4 and associated with poor clinical prognosis. LINC01134 can promote Nrf2 recruitment to the GPX4 promoter region *via* the interaction of the RNA-DNA sequence to increase the transcripts of GPX4, and while LINC01134 silencing leads to oxaliplatin sensitivity by inhibiting the total ROS, lipid ROS, and MDA levels and decreasing the GSH/GSSG ratio (Kang et al., 2022). Guanghao Li et al. predicted some ferroptosis-related lncRNAs associating with immunosuppressive phenotype and drug sensitivity (Li et al., 2022b). Moreover, in Liu, Z. et al.’s research, circRNA cIARS were found to be upregulated in sorafenib-induced HCC cells, which can promote ferroptosis *via* ferritinophagy by repressing the anti-autophagy effect of ALKBH5 (Liu et al., 2020). CircIL4R was also found to inhibit ferroptosis in the HCC, which can act as a sponge of miR-541-3p, affecting the expression of GPX4 (Xu et al., 2020). Another study found that circ0097009 was increased in HCC tissues and cell lines. Circ0097009 knockdown promoted ferroptosis *via* the circ0097009/miR-1261/SLC7A11 axis (Lyu et al., 2021) (Figure 2F). It seems that ncRNAs participate in multiple steps of ferroptosis *via* regulating key genes or pathways. HCC is a promising tumor that may benefit from ferroptosis-related therapies.

### 2.7 Renal cell carcinoma

More than 431,000 kidney cancer cases and 179,000 deaths occurred worldwide in 2020 (Sung et al., 2021). Recent research reports that decreased miR-4735-3p is verified in clear cell renal cell carcinoma (RCC) tissues. Overexpressed miR-4735-3p can downregulate SLC40A1 to facilitate ferroptosis (Zhu et al., 2022). In addition, icariside II (an antitumor flavonoid) increased miR-324-3p, which can reduce GPX4 expression, and facilitated the ferroptosis of RCC cells (Yu et al., 2022) (Figure 2G). In short, there is still insufficient research to clarify the relationship between ncRNAs and ferroptosis pathways. Therefore, the importance of ferroptosis-related ncRNAs in the progression of RCC is not yet determined.

### 2.8 Bladder cancer

There were approximately 573,000 new cases and 213,000 deaths in bladder cancer in 2020 globally, and the incidence rate in males is approximately four times that in females (Sung et al., 2021). It is reported that lncRNA RP11-89 functions as a sponge for miR-129-5p, which targets PROM2. PROM2 induces the formation of multivesicular bodies to promote iron export and ferroptosis resistance (Luo et al., 2021). In addition, integrated analyses predicted that some lncRNAs are closely related with prognosis in bladder cancer (Chen et al., 2021a; Wang et al., 2022d). Wang, L. et al. suggested that circST6GALNAC6 functions as a tumor suppressor in bladder cancer, which can facilitate ferroptosis *via* the circST6GALNAC6/HSPB1/p38 MAPK axis. Meanwhile, circST6GALNAC6 can suppress small heat shock protein 1 (HSPB1) by binding to the N-terminus to block the phosphorylation of HSPB1 at the Ser-15 site, thus activating the P38 MAPK pathway (Wang et al., 2022a) (Figure 2H). The current research only shows that ncRNAs affect ferroptosis through the PROM2 and MAPK pathways, and the relationship between ncRNAs and other key genes/pathways is unclear. Therefore, more research is expected concerning the bladder cancer phenotype mediated by ncRNAs through ferroptosis.

### 2.9 Prostate cancer

Prostate cancer is the second most common cancer among males, and there were nearly 375,000 deaths of prostate cancer patients worldwide in 2020 (Wang et al., 2021b; Sung et al., 2021). Recent research suggests that miR-15a represses cell proliferation and facilitates ferroptosis by binding to GPX4’s 3’UTR in prostate cancer (Xu et al., 2022a). Moreover, Jiang, X. et al. uncovered that lncRNA PCAT1 overexpression suppresses ferroptosis and promotes docetaxel resistance, while its knockdown has the opposite effect. On one hand, lncRNA PCAT1 elevates C-Myc protein stability *via* interacting with its 151-202 aa, thus upregulating SLC7A11 by binding to its promoter at the transcription level. On the other hand, lncRNA PCAT1 increases SLC7A11 by sponging miR-25-3p. Moreover, transcription factor TFAP2C can bind to the PCAT1 promoter to elevate its expression (Jiang et al., 2022a). In Zhang, Y.’s research, lncRNA OIP5-AS1 inhibited ferroptosis and facilitated cell growth, colony formation, and cell invasion in PC3 and DU145 exposed to cadmium. LncRNA OIP5-AS1 functions as a ceRNA for miR-128-3p and represses ferroptosis *via* the miR-128-3p/SLC7A11 pathway (Zhang et al., 2021c) (Figure 2I). Compared with other urinary tumors, ncRNAs in prostate cancer seems to be more inclined to affect classic genes related to ferroptosis, such as GPX4 and SLC7A11, which means that experimental design from the downstream classic gene is a good option for the study of ferroptosis-related ncRNAs.

### 2.10 Breast cancer

Breast cancer has become the main cause of cancer-related deaths globally. There were 2.3 million new diagnosed breast cancer cases in 2020, accounting for 11.7% of all cancer cases (Sung et al., 2021). In studies on ferroptosis-related ncRNAs, Yadav, P. et al. found that miR-5096 targets SLC7A11 to facilitate ferroptosis in MDA-MB-231 cells, thus inhibiting proliferation, colony formation, migration, and invasion (Yadav et al., 2021). Metformin, a hypoglycemic drug, promotes ferroptosis through miR-324-3p, which downregulates GPX4 by binding to its 3’UTR (Hou et al., 2021). CircRHOT1 notably inhibits ferroptosis and the levels of ROS, iron in breast cancer cells through sponging miR-106a-5p, which targets the signal transducer and activator of transcription 3 (STAT3) (Zhang et al., 2021b). Bazhabayi, M.’s work showed that upregulated circGFRA1 was clarified in HER2+ breast cancer tissues and cells, while circGFRA1 knockdown induced ferroptosis and inhibited cell proliferation and metastasis in HER2+ breast cancer cells. Mechanically, circGFRA1 can sponge miR-1228, which targets AIFM2 (a ferroptosis suppressor) (Bazhabayi et al., 2021) (Figure 2J). Taken together, STAT3 and AIFM2 are two unfamiliar ferroptosis-related regulators in breast cancer, suggesting that novel ferroptosis-related signals mediated by ncRNAs may be more prevailing in breast cancer.

### 2.11 Ovarian cancer and cervical cancer

There were 314,000 (3.4% of all new cancer cases) new ovarian cancer cases and 207,000 deaths (4.7% of all deaths) globally in 2020 (Sung et al., 2021). Recent studies found that the upregulation of miR-424-5p suppresses ACSL4 by directly binding to its 3’-UTR, which subsequently reduces erastin- and RSL3-induced ferroptosis in ovarian cancer (Ma et al., 2021b). Li Cai demonstrated that lncRNA ADAMTS9-AS1 attenuates ferroptosis by targeting the miR-587/SLC7A11 axis (Cai et al., 2022) (Figure 2K). There is little evidence identifying the regulatory role of ferroptosis mediated by ncRNAs. Therefore, more studies on the molecular mechanisms of ncRNA-regulated ferroptosis need to be conducted.

Cervical cancer is the fourth cause of cancer deaths among females. It is estimated that 604,000 new cases and 342,000 deaths occurred globally in 2020 (Sung et al., 2021). In previous research on ferroptosis-related circRNAs, Ou, R.’s team identified decreased circLMO1 in cervical cancer cell lines. CircLMO1 suppresses cell proliferation and invasion *via* sponging miR-4291 to upregulate ACSL4, thus promoting ferroptosis. Interestingly, they also found that I3RC (reverse complementary sequence in intron 3) can accelerate the circularization of circLMO1, and RNA-binding protein DExH-Box Helicase 9 (DHX9) negatively regulates circLMO1 (Ou et al., 2022). Wu, P. et al. showed that the circEPSTI1-miR-375/409-3P/515-5p-SLC7A11 axis affects the proliferation of cervical cancer *via* the competing endogenous RNAs (ceRNAs) mechanism, and circEPSTI1 silence promotes the ferroptosis process mediated by SLC7A11 (Wu et al., 2021b). Similarly, Liu, Y. et al. suggest that circACAP2 silence suppresses cell proliferation and promotes ferroptosis in SiHa and HeLa cells. CircACAP2 functions as a ceRNA of miR-193a-5p, thus regulating its target GPX4 (Liu et al., 2022b) (Figure 2K). Various ferroptosis-associated traditional signals such as SLC7A11, GPX4, and ACSL4 have been uncovered in cervical cancer, and it seems that circRNAs provide prominence to regulate ferroptosis-related signals in cervical cancer. Whether circRNAs can develop into therapeutic targets to treat breast cancer needs further research.

### 2.12 Acute myeloid leukemia

More than 474,000 new leukemia cases and 311,000 deaths (3.1% of all deaths in both sexes) occurred worldwide in 2020 (Sung et al., 2021). LINC00618 attenuates the expression of lymphoid-specific helicase (LSH), and LSH can increase the transcription of SLC7A11 after recruitment to the promoter regions of SLC7A11, further promoting ferroptosis in human acute myeloid leukemia (AML). Interestingly, LINC00618 can be induced by the chemotherapeutic reagent vincristine (VCR) (Wang et al., 2021c). In addition, decreased circKDM4C is found in AML patients, while circKDM4C overexpression can promote ferroptosis and repress cell proliferation, migration, and invasion. Mechanically, circKDM4C functions as a sponge for hsa-let-7b-5p to increase p53 expression (Dong et al., 2021) (Figure 2L). The regulation of ncRNAs involved with ferroptosis in AML is still insufficient, and more studies involving in-depth exploration need to be conducted.

### 2.13 Glioblastoma

Glioblastoma is characterized by malignant cell heterogeneity, invasion and migration, which leads to its incurability (Venkataramani et al., 2022). Xu, P.’s team suggested that the miR-147a mimic inhibits cell survival and induces ferroptosis by targeting SLC40A1’s 3’UTR in U87MG and A172 cells but not iron storage protein FTH1 or intake protein TFR (Xu et al., 2022b). Bao, C. uncovered that miR-670-3p inhibits ferroptosis by targeting ACSL4 in U87MG and A172 cells (Bao et al., 2021). Chen, Q. et al. found that lncRNA TMEM161B-AS1 knockdown induces ferroptosis, thus repressing cell proliferation, migration, and invasion in U87 and U251 cells. Mechanically, lncRNA TMEM161B-AS1 sponges hsa-miR-27a-3p, which targets FANCD2 and CD44 *via* binding to the seed region of their 3’ UTR sequence. FANCD2 and CD44 silencing can increase intracellular iron accumulation and lipid ROS (Chen et al., 2021b). CircLRFN5 binds to transcription factor PRRX2 and promotes its degradation *via* a ubiquitin-mediated proteasomal pathway. PRRX2 can transcriptionally upregulate GCH1 expression in GSCs, which is a ferroptosis suppressor *via* generating the antioxidant tetrahydrobiopterin (BH4) (Jiang et al., 2022b) (Figure 2M). Taken together, ncRNAs participate in the regulation of iron transporter SLC40A1 and PRRX2 degradation in GBM. However, more studies are expected to explore the regulatory mechanism of ncRNAs in glioblastoma through other ferroptosis molecules, such as GPX4, SLC7A11, and Nrf2.

### 2.14 Melanoma

More than 324,000 new diagnosed melanoma cases and 57,000 deaths occurred worldwide in 2020 (Sung et al., 2021). Zhang, K. et al. reported that miR-9 inhibits ferroptosis by downregulating glutamic-oxaloacetic transaminase (GOT1) (Zhang et al., 2018b). In addition, miR-137 inhibits ferroptosis by targeting SLC1A5 in A375 and G-361 melanoma cells in a glutaminolysis-dependent manner, thus inhibiting Gln uptake, while SLC1A5 overexpression can reverse the suppression effect on ferroptosis (Luo et al., 2018). Liao, Y.’s team found that miR-130b-3p suppresses ferroptosis by binding to the 3’UTR of DKK1 and regulating DKK1-dependent Nrf2/HO-1 signaling in A375 and G-361 cells. MiR-130b-3p suppresses intracellular iron accumulation and lipid ROS, while DKK1 overexpression can block the suppression of miR-130b-3p (Liao et al., 2021). Ferroptosis is also involved in enhancing chemotherapy efficacy. Guo, W. et al. found that miR-21-3p promotes ferroptosis and lipid peroxidation by targeting TXNRD1. Moreover, the miR-21-3p/TXNRD1 axis also increases sensitivity to anti-PD-1 immunotherapy by inducing ferroptosis in melanoma (Guo et al., 2022) (Figure 2N). Different from other cancers, ncRNAs mainly mediate the new system Xc^−^ subunit SLC1A5, which provides us with another dimension of thought concerning ferroptosis regulation and a new perspective on melanoma inhibition and chemotherapy sensitivity through ferroptosis.

## 3 Discussion

As a distinctive PCD regulating cancer development, ferroptosis is characterized by an abnormal iron metabolism and lipid peroxidation. A large majority of studies suggest that ferroptosis is closely associated with malignant cancer development. Many familiar ferroptosis-remarked molecules have been confirmed, including system Xc^−^, GPX4, ACSL4, Keap1/Nrf2 signals, and p53. Nevertheless, there are no ferroptosis-remarked ncRNAs yet. Some ncRNAs regulate ferroptosis in different malignant cancers; for example, miR-324-3p mediates ferroptosis in RCC and breast cancer simultaneously (Hou et al., 2021; Yu et al., 2022), and miR-27a-3p regulates ferroptosis in lung cancer and glioblastoma (Chen et al., 2021b; Lu et al., 2021b). However, it has not been determined whether miR-324-3p or miR-27a-3p mediates the ferroptosis process in other malignant cancers. Therefore, more research is expected to clarify ncRNAs’ commonality effectiveness in ferroptosis among cancers. Some unfamiliar ferroptosis markers have also been validated, such as STAT3, AIFM2, and VDAC3.

A large number of studies have shown that ncRNAs can affect the progression of liver cancer by mediating various ferroptosis-related classical molecules or signals, such as SLC7A11, GPX4, and ACSL4 (Bai et al., 2020; Zhang et al., 2022b; Lu et al., 2022). However, there are few reports on pancreatic cancer and acute myeloid leukemia. On one hand, it may be due to the bias of the researcher’s study. On the other hand, we suppose that different types of cancer cells have discrepant sensitivity to ferroptosis. We believe that ncRNA-mediated tumor progression through ferroptosis is possibly individual in different malignant cancers. Current studies still suggest that miR-4443, lncRNA BDNF-AS, and circRNA-ST6GALNAC6 play indispensable roles in a variety of malignant cancers, and they may become potential markers for the diagnosis and treatment of some specific cancers (Song et al., 2021; Wang et al., 2022a; Huang et al., 2022).

There are few reports on the upstream regulation of ferroptosis-related ncRNAs until now. We believe the conventional transcription or splicing factors can potentially reveal a hidden repertoire as a mechanism of exploration. For example, HIF-1α can bind to the endogenous HRE site in the lncRNA PMAN promoter to increase lncRNA PMAN (Lin et al., 2022). ETS1 can bind to the miR-23a-3p promoter motif to stimulate its expression (Lu et al., 2022). Transcription factor binding to IGHM enhancer 3 (TFE3) can be recruited to the LINC01606 promoter to promote LINC01606 transcription (Luo et al., 2022). p53 can also bind to the lncRNA NEAT1 promoter to elevate its expression (Zhang et al., 2022c). It is reported that the NF-kB pathway can promote tumorigenesis by inducing miR-130b/301b (Man et al., 2019), but there is still a lack of systematic research on the upstream of ferroptosis-related ncRNAs, and our next work will focus on this discovery and attempt to discover some common characteristics in order to determine upstream regulatory mechanisms.

Targeting ncRNA-mediated ferroptosis may provide a prognosis reference and novel therapeutic methods to repress the development of malignant tumors. On one hand, an increasing number of studies suggest that ncRNAs can function as a biomarker to predict tumor progression and clinical prognosis. On the other hand, according to studies on ncRNA-regulated ferroptosis, the ferroptosis process can be altered *via* the intervention of the expression of ncRNAs, thus affecting cancer cell proliferation, invasion, migration, and chemoresistance. However, there are possibly many limitations in the application of potential ncRNA-targeted therapeutic methods. First, the ncRNA-mediated ferroptosis process is finite in the regulation of tumorigenesis. Second, the individual differences in ncRNA gene expression and the individual sensibility to intervention compounds are also two unpredictable directions. Third, it is complicated for ncRNAs to regulate ferroptosis between cancer progression and chemoresistance. Consequently, more studies need to be performed to explore clinical compounds targeting ferroptosis-related ncRNAs.

## References

[B1] Bai T. Liang R. Zhu R. Wang W. Zhou L. Sun Y. (2020). MicroRNA-214-3p enhances erastin-induced ferroptosis by targeting ATF4 in hepatoma cells. J. Cell Physiol. 235 (7-8), 5637–5648. 10.1002/jcp.29496 31960438

[B2] Bao C. Zhang J. Xian S. Y. Chen F. (2021). MicroRNA-670-3p suppresses ferroptosis of human glioblastoma cells through targeting ACSL4. Free Radic. Res. 55 (7), 853–864. 10.1080/10715762.2021.1962009 34323631

[B3] Bazhabayi M. Qiu X. Li X. Yang A. Wen W. Zhang X. (2021). CircGFRA1 facilitates the malignant progression of HER-2-positive breast cancer via acting as a sponge of miR-1228 and enhancing AIFM2 expression. J. Cell Mol. Med. 25 (21), 10248–10256. 10.1111/jcmm.16963 34668628 PMC8572792

[B4] Belaidi A. A. Bush A. I. (2016). Iron neurochemistry in alzheimer's disease and Parkinson's disease: Targets for therapeutics. J. Neurochem. 139 (1), 179–197. 10.1111/jnc.13425 26545340

[B5] Bi G. Liang J. Zhao M. Zhang H. Jin X. Lu T. (2022). miR-6077 promotes cisplatin/pemetrexed resistance in lung adenocarcinoma via CDKN1A/cell cycle arrest and KEAP1/ferroptosis pathways. Mol. Ther. Nucleic Acids 28, 366–386. 10.1016/j.omtn.2022.03.020 35505963 PMC9035384

[B6] Brandes R. P. Weissmann N. Schröder K. (2014). Nox family NADPH oxidases: Molecular mechanisms of activation. Free Radic. Biol. Med. 76, 208–226. 10.1016/j.freeradbiomed.2014.07.046 25157786

[B7] Brown C. W. Amante J. J. Chhoy P. Elaimy A. L. Liu H. Zhu L. J. (2019). Prominin2 drives ferroptosis resistance by stimulating iron export. Dev. Cell 51 (5), 575–586. 10.1016/j.devcel.2019.10.007 31735663 PMC8316835

[B8] Cai L. Hu X. Ye L. Bai P. Jie Y. Shu K. (2022). Long non-coding RNA ADAMTS9-AS1 attenuates ferroptosis by Targeting microRNA-587/solute carrier family 7 member 11 axis in epithelial ovarian cancer. Bioengineered 13 (4), 8226–8239. 10.1080/21655979.2022.2049470 35311457 PMC9161843

[B9] Chen D. Fan Z. Rauh M. Buchfelder M. Eyupoglu I. Y. SavaskaN N. (2017). ATF4 promotes angiogenesis and neuronal cell death and confers ferroptosis in a xCT-dependent manner. Oncogene 36 (40), 5593–5608. 10.1038/onc.2017.146 28553953 PMC5633655

[B10] Chen D. Tavana O. Chu B. Erber L. Chen Y. Baer R. (2017). NRF2 is a major target of ARF in p53-independent tumor suppression. Mol. Cell 68 (1), 224–232. 10.1016/j.molcel.2017.09.009 28985506 PMC5683418

[B11] Chen M. Nie Z. Li Y. Gao Y. Wen X. Cao H. (2021). A new ferroptosis-related lncRNA signature predicts the prognosis of bladder cancer patients. Front. Cell Dev. Biol. 9, 699804. 10.3389/fcell.2021.699804 34869304 PMC8635160

[B12] Chen Q. Wang W. Wu Z. Chen S. Chen X. Zhuang S. (2021). Over-expression of lncRNA TMEM161B-AS1 promotes the malignant biological behavior of glioma cells and the resistance to temozolomide via up-regulating the expression of multiple ferroptosis-related genes by sponging hsa-miR-27a-3p. Cell Death Discov. 7 (1), 311. 10.1038/s41420-021-00709-4 34689169 PMC8542043

[B13] Circu M. L. Aw T. Y. (2008). Glutathione and apoptosis. Free Radic. Res. 42 (8), 689–706. 10.1080/10715760802317663 18671159 PMC3171829

[B14] Dai T. Li J. Lu X. Ye L. Yu H. Zhang L. (2021). Prognostic role and potential mechanisms of the ferroptosis-related metabolic gene signature in hepatocellular carcinoma. Pharmgenomics Pers. Med. 14, 927–945. 10.2147/PGPM.S319524 34377010 PMC8349220

[B15] de Carvalho C. Caramujo M. J. (2018). The various roles of fatty acids. Molecules 23 (10), 2583. 10.3390/molecules23102583 30304860 PMC6222795

[B16] Dixon S. J. Lemberg K. M. Lamprecht M. R. Skouta R. Zaitsev E. M. Gleason C. E. (2012). Ferroptosis: An iron-dependent form of nonapoptotic cell death. Cell 149 (5), 1060–1072. 10.1016/j.cell.2012.03.042 22632970 PMC3367386

[B17] Dixon S. J. Patel D. N. Welsch M. Skouta R. Lee E. D. Hayano M. (2014). Pharmacological inhibition of cystine-glutamate exchange induces endoplasmic reticulum stress and ferroptosis. Elife 3, e02523. 10.7554/eLife.02523 24844246 PMC4054777

[B18] Dixon S. J. Stockwell B. R. (2014). The role of iron and reactive oxygen species in cell death. Nat. Chem. Biol. 10 (1), 9–17. 10.1038/nchembio.1416 24346035

[B19] Dong L. H. Huang J. J. Zu P. Liu J. Gao X. Du J. W. (2021). CircKDM4C upregulates P53 by sponging hsa-let-7b-5p to induce ferroptosis in acute myeloid leukemia. Environ. Toxicol. 36 (7), 1288–1302. 10.1002/tox.23126 33733556

[B20] Donovan A. Lima C. A. Pinkus J. L. Pinkus G. S. Zon L. I. Robine S. (2005). The iron exporter ferroportin/Slc40a1 is essential for iron homeostasis. Cell Metab. 1 (3), 191–200. 10.1016/j.cmet.2005.01.003 16054062

[B21] El Hout M. Dos Santos L. Hamai A. Mehrpour M. (2018). A promising new approach to cancer therapy: Targeting iron metabolism in cancer stem cells. Semin. Cancer Biol. 53, 125–138. 10.1016/j.semcancer.2018.07.009 30071257

[B22] Fan H. Ai R. Mu S. Niu X. Guo Z. Liu L. (2022). MiR-19a suppresses ferroptosis of colorectal cancer cells by targeting IREB2. Bioengineered 13 (5), 12021–12029. 10.1080/21655979.2022.2054194 35599631 PMC9275930

[B23] Fan Z. Wirth A. K. Chen D. Wruck C. J. Rauh M. Buchfelder M. (2017). Nrf2-Keap1 pathway promotes cell proliferation and diminishes ferroptosis. Oncogenesis 6 (8), e371. 10.1038/oncsis.2017.65 28805788 PMC5608917

[B24] Florean C. Song S. Dicato M. Diederich M. (2019). Redox biology of regulated cell death in cancer: A focus on necroptosis and ferroptosis. Free Radic. Biol. Med. 134, 177–189. 10.1016/j.freeradbiomed.2019.01.008 30639617

[B25] Fuchs Y. Steller H. (2011). Programmed cell death in animal development and disease. Cell 147 (4), 742–758. 10.1016/j.cell.2011.10.033 22078876 PMC4511103

[B26] Galluzzi L. Vitale I. Aaronson S. A. Abrams J. M. Adam D. Agostinis P. (2018). Molecular mechanisms of cell death: Recommendations of the nomenclature committee on cell death 2018. Cell Death Differ. 25 (3), 486–541. 10.1038/s41418-017-0012-4 29362479 PMC5864239

[B27] Gao M. Monian P. Pan Q. Zhang W. Xiang J. Jiang X. (2016). Ferroptosis is an autophagic cell death process. Cell Res. 26 (9), 1021–1032. 10.1038/cr.2016.95 27514700 PMC5034113

[B28] Gao M. Monian P. Quadri N. Ramasamy R. Jiang X. (2015). Glutaminolysis and transferrin regulate ferroptosis. Mol. Cell 59 (2), 298–308. 10.1016/j.molcel.2015.06.011 26166707 PMC4506736

[B29] Gaschler M. M. Andia A. A. Liu H. Csuka J. M. Hurlocker B. Vaiana C. A. (2018). FINO(2) initiates ferroptosis through GPX4 inactivation and iron oxidation. Nat. Chem. Biol. 14 (5), 507–515. 10.1038/s41589-018-0031-6 29610484 PMC5899674

[B30] Geng H. Qian R. Zhang L. Yang C. Xia X. Wang C. (2022). Clinical outcomes and potential therapies prediction of subgroups based on a ferroptosis-related long non-coding RNA signature for gastric cancer. Aging (Albany NY) 14 (15), 6358–6376. 10.18632/aging.204227 35969182 PMC9417219

[B31] Gomaa A. Peng D. Chen Z. Soutto M. Abouelezz K. Corvalan A. (2019). Epigenetic regulation of AURKA by miR-4715-3p in upper gastrointestinal cancers. Sci. Rep. 9 (1), 16970. 10.1038/s41598-019-53174-6 31740746 PMC6861278

[B32] Guan L. Wang F. Wang M. Han S. Cui Z. Xi S. (2022). Downregulation of HULC induces ferroptosis in hepatocellular carcinoma via targeting of the miR-3200-5p/ATF4 Axis. Oxid. Med. Cell Longev. 2022, 9613095. 10.1155/2022/9613095 35615577 PMC9126659

[B33] Guan X. Li X. Yang X. Yan J. Shi P. Ba L. (2019). The neuroprotective effects of carvacrol on ischemia/reperfusion-induced hippocampal neuronal impairment by ferroptosis mitigation. Life Sci. 235, 116795. 10.1016/j.lfs.2019.116795 31470002

[B34] Guo W. Wu Z. Chen J. Guo S. You W. Wang S. (2022). Nanoparticle delivery of miR-21-3p sensitizes melanoma to anti-PD-1 immunotherapy by promoting ferroptosis. J. Immunother. Cancer 10 (6), e004381. 10.1136/jitc-2021-004381 35738798 PMC9226924

[B35] Guo Y. Qu Z. Bai F. Xing J. Ding Q. Zhou J. (2021). Identification of a prognostic ferroptosis-related lncRNA signature in the tumor microenvironment of lung adenocarcinoma. Cell Death Discov. 7 (1), 190. 10.1038/s41420-021-00576-z 34312372 PMC8313561

[B36] Han Y. Gao X. Wu N. Jin Y. Zhou H. Wang W. (2022). Long noncoding RNA LINC00239 inhibits ferroptosis in colorectal cancer by binding to Keap1 to stabilize Nrf2. Cell Death Dis. 13 (8), 742. 10.1038/s41419-022-05192-y 36038548 PMC9424287

[B37] Higdon A. Diers A. R. Oh J. Y. Landar A. Darley-Usmar V. M. (2012). Cell signalling by reactive lipid species: New concepts and molecular mechanisms. Biochem. J. 442 (3), 453–464. 10.1042/BJ20111752 22364280 PMC3286857

[B38] Hou W. Xie Y. Song X. Sun X. Lotze M. T. Zeh H. J. (2016). Autophagy promotes ferroptosis by degradation of ferritin. Autophagy 12 (8), 1425–1428. 10.1080/15548627.2016.1187366 27245739 PMC4968231

[B39] Hou Y. Cai S. Yu S. Lin H. (2021). Metformin induces ferroptosis by targeting miR-324-3p/GPX4 axis in breast cancer. Acta Biochim. Biophys. Sin. (Shanghai) 53 (3), 333–341. 10.1093/abbs/gmaa180 33522578

[B40] Huang G. Xiang Z. Wu H. He Q. Dou R. Lin Z. (2022). The lncRNA BDNF-AS/WDR5/FBXW7 axis mediates ferroptosis in gastric cancer peritoneal metastasis by regulating VDAC3 ubiquitination. Int. J. Biol. Sci. 18 (4), 1415–1433. 10.7150/ijbs.69454 35280682 PMC8898362

[B41] Imai H. Nakagawa Y. (2003). Biological significance of phospholipid hydroperoxide glutathione peroxidase (PHGPx, GPx4) in mammalian cells. Free Radic. Biol. Med. 34 (2), 145–169. 10.1016/s0891-5849(02)01197-8 12521597

[B42] Jiang L. Kon N. Li T. Wang S. J. Su T. Hibshoosh H. (2015). Ferroptosis as a p53-mediated activity during tumour suppression. Nature 520 (7545), 57–62. 10.1038/nature14344 25799988 PMC4455927

[B43] Jiang X. Guo S. Xu M. Ma B. Liu R. Xu Y. (2022). TFAP2C-Mediated lncRNA PCAT1 inhibits ferroptosis in docetaxel-resistant prostate cancer through c-Myc/miR-25-3p/SLC7A11 signaling. Front. Oncol. 12, 862015. 10.3389/fonc.2022.862015 35402284 PMC8985761

[B44] Jiang X. Yuan Y. Tang L. Wang J. Zhang D. Duan L. (2021). Systematic analysis and validation of the prognosis, immunological role and biology function of the ferroptosis-related lncRNA GSEC/miRNA-101-3p/CISD1 Axis in lung adenocarcinoma. Front. Mol. Biosci. 8, 793732. 10.3389/fmolb.2021.793732 35320929 PMC8936422

[B45] Jiang Y. Zhao J. Li R. Liu Y. Zhou L. Wang C. (2022). CircLRFN5 inhibits the progression of glioblastoma via PRRX2/GCH1 mediated ferroptosis. J. Exp. Clin. Cancer Res. 41 (1), 307. 10.1186/s13046-022-02518-8 36266731 PMC9583503

[B46] Kagan V. E. Mao G. Qu F. Angeli J. P. F. Doll S. Croix C. S. (2017). Oxidized arachidonic and adrenic PEs navigate cells to ferroptosis. Nat. Chem. Biol. 13 (1), 81–90. 10.1038/nchembio.2238 27842066 PMC5506843

[B47] Kang X. Huo Y. Jia S. He F. Li H. Zhou Q. (2022). Silenced LINC01134 enhances oxaliplatin sensitivity by facilitating ferroptosis through GPX4 in hepatocarcinoma. Front. Oncol. 12, 939605. 10.3389/fonc.2022.939605 35875091 PMC9304856

[B48] Kerins M. J. Ooi A. (2018). The roles of NRF2 in modulating cellular iron homeostasis. Antioxid. Redox Signal 29 (17), 1756–1773. 10.1089/ars.2017.7176 28793787 PMC6208163

[B49] Kobayashi A. Kang M. I. Okawa H. Ohtsuji M. Zenke Y. Chiba T. (2004). Oxidative stress sensor Keap1 functions as an adaptor for Cul3-based E3 ligase to regulate proteasomal degradation of Nrf2. Mol. Cell Biol. 24 (16), 7130–7139. 10.1128/MCB.24.16.7130-7139.2004 15282312 PMC479737

[B50] Küch E. M. Vellaramkalayil R. Zhang I. Lehnen D. Brugger B. Sreemmel W. (2014). Differentially localized acyl-CoA synthetase 4 isoenzymes mediate the metabolic channeling of fatty acids towards phosphatidylinositol. Biochim. Biophys. Acta 1841 (2), 227–239. 10.1016/j.bbalip.2013.10.018 24201376

[B51] Lewerenz J. Hewett S. J. Huang Y. Lambros M. Gout P. W. Kalivas P. W. (2013). The cystine/glutamate antiporter system x(c)(-) in health and disease: From molecular mechanisms to novel therapeutic opportunities. Antioxid. Redox Signal 18 (5), 522–555. 10.1089/ars.2011.4391 22667998 PMC3545354

[B52] Li G. Liu Y. Zhang Y. Xu Y. Zhang J. Wei X. (2022). A novel ferroptosis-related long non-coding RNA prognostic signature correlates with genomic heterogeneity, immunosuppressive phenotype, and drug sensitivity in hepatocellular carcinoma. Front. Immunol. 13, 929089. 10.3389/fimmu.2022.929089 35874689 PMC9304774

[B54] Li J. Xiang R. Song W. Wu J. Kong C. Fu T. (2022). A novel ferroptosis-related LncRNA pair prognostic signature predicts immune landscapes and treatment responses for gastric cancer patients. Front. Genet. 13, 899419. 10.3389/fgene.2022.899419 35795206 PMC9250987

[B55] Liao Y. Jia X. Ren Y. Deji Z. Gesang Y. Ning N. (2021). Suppressive role of microRNA-130b-3p in ferroptosis in melanoma cells correlates with DKK1 inhibition and Nrf2-HO-1 pathway activation. Hum. Cell 34 (5), 1532–1544. 10.1007/s13577-021-00557-5 34117611

[B56] Lin Z. Song J. Gao Y. Huang S. Dou R. Zhong P. (2022). Hypoxia-induced HIF-1α/lncRNA-PMAN inhibits ferroptosis by promoting the cytoplasmic translocation of ELAVL1 in peritoneal dissemination from gastric cancer. Redox Biol. 52, 102312. 10.1016/j.redox.2022.102312 35447413 PMC9043498

[B57] Liu L. Yao H. Zhou X. Chen J. Chen G. Shi X. (2022). MiR-15a-3p regulates ferroptosis via targeting glutathione peroxidase GPX4 in colorectal cancer. Mol. Carcinog. 61 (3), 301–310. 10.1002/mc.23367 34727409

[B58] Liu Y. Dou M. Song X. Dong Y. Liu S. Liu H. (2019). The emerging role of the piRNA/piwi complex in cancer. Mol. Cancer 18 (1), 123. 10.1186/s12943-019-1052-9 31399034 PMC6688334

[B59] Liu Y. Li L. Yang Z. Wen D. Hu Z. (2022). Circular RNA circACAP2 suppresses ferroptosis of cervical cancer during malignant progression by miR-193a-5p/GPX4. J. Oncol. 2022, 5228874. 10.1155/2022/5228874 35847361 PMC9286899

[B60] Liu Z. Wang Q. Wang X. Xu Z. Wei X. Li J. (2020). Circular RNA cIARS regulates ferroptosis in HCC cells through interacting with RNA binding protein ALKBH5. Cell Death Discov. 6, 72. 10.1038/s41420-020-00306-x 32802409 PMC7414223

[B61] Lu L. Liu L. P. Zhao Q. Q. Gui R. Zhao Q. Y. (2021). Identification of a ferroptosis-related LncRNA signature as a novel prognosis model for lung adenocarcinoma. Front. Oncol. 11, 675545. 10.3389/fonc.2021.675545 34249715 PMC8260838

[B62] Lu X. Kang N. Ling X. Pan M. Du W. Gao S. (2021). MiR-27a-3p promotes non-small cell lung cancer through slc7a11-mediated-ferroptosis. Front. Oncol. 11, 759346. 10.3389/fonc.2021.759346 34722314 PMC8548660

[B63] Lu Y. Chan Y. T. Tan H. Y. Zhang C. Guo W. Xu Y. (2022). Epigenetic regulation of ferroptosis via ETS1/miR-23a-3p/ACSL4 axis mediates sorafenib resistance in human hepatocellular carcinoma. J. Exp. Clin. Cancer Res. 41 (1), 3. 10.1186/s13046-021-02208-x 34980204 PMC8722264

[B64] Lu Y. Qin H. Jiang B. Lu W. Hao J. Cao W. (2021). KLF2 inhibits cancer cell migration and invasion by regulating ferroptosis through GPX4 in clear cell renal cell carcinoma. Cancer Lett. 522, 1–13. 10.1016/j.canlet.2021.09.014 34520818

[B65] Luo M. Wu L. Zhang K. Wang H. Zhang T. Gutierrez L. (2018). miR-137 regulates ferroptosis by targeting glutamine transporter SLC1A5 in melanoma. Cell Death Differ. 25 (8), 1457–1472. 10.1038/s41418-017-0053-8 29348676 PMC6113319

[B66] Luo W. Wang J. Xu W. Ma C. Wan F. Huang Y. (2021). LncRNA RP11-89 facilitates tumorigenesis and ferroptosis resistance through PROM2-activated iron export by sponging miR-129-5p in bladder cancer. Cell Death Dis. 12 (11), 1043. 10.1038/s41419-021-04296-1 34728613 PMC8563982

[B67] Luo Y. Huang S. Wei J. Zhou H. Wang W. Yang J. (2022). Long noncoding RNA LINC01606 protects colon cancer cells from ferroptotic cell death and promotes stemness by SCD1-Wnt/β-catenin-TFE3 feedback loop signalling. Clin. Transl. Med. 12 (4), e752. 10.1002/ctm2.752 35485210 PMC9052012

[B68] Lyu N. Zeng Y. Kong Y. Chen Q. Deng H. Ou S. (2021). Ferroptosis is involved in the progression of hepatocellular carcinoma through the circ0097009/miR-1261/SLC7A11 axis. Ann. Transl. Med. 9 (8), 675. 10.21037/atm-21-997 33987373 PMC8106082

[B69] Ma L. L. Liang L. Zhou D. Wang S. W. (2021). Tumor suppressor miR-424-5p abrogates ferroptosis in ovarian cancer through targeting ACSL4. Neoplasma 68 (1), 165–173. 10.4149/neo_2020_200707N705 33038905

[B70] Ma L. Zhang X. Yu K. Xu X. Chen T. Shi Y. (2021). Targeting SLC3A2 subunit of system X(C)(-) is essential for m(6)A reader YTHDC2 to be an endogenous ferroptosis inducer in lung adenocarcinoma. Free Radic. Biol. Med. 168, 25–43. 10.1016/j.freeradbiomed.2021.03.023 33785413

[B71] Maiorino M. Conrad M. Ursini F. (2018). GPx4, lipid peroxidation, and cell death: Discoveries, rediscoveries, and open issues. Antioxid. Redox Signal 29 (1), 61–74. 10.1089/ars.2017.7115 28462584

[B72] Man X. Piao C. Kong C. Cui X. Jiang Y. (2019). USP13 functions as a tumor suppressor by blocking the NF-kB-mediated PTEN downregulation in human bladder cancer. J. Exp. Clin. Cancer Res. 38 (1), 259. 10.1186/s13046-019-1262-4 31200745 PMC6570860

[B73] Mao C. Wang X. Liu Y. Wang M. Yan B. Jiang Y. (2018). A G3BP1-interacting lncRNA promotes ferroptosis and apoptosis in cancer via nuclear sequestration of p53. Cancer Res. 78 (13), 3484–3496. 10.1158/0008-5472.CAN-17-3454 29588351 PMC8073197

[B74] Ni H. Qin H. Sun C. Liu Y. Ruan G. Guo Q. (2021). MiR-375 reduces the stemness of gastric cancer cells through triggering ferroptosis. Stem Cell Res. Ther. 12 (1), 325. 10.1186/s13287-021-02394-7 34090492 PMC8180146

[B75] Nishizawa H. Matsumoto M. Shindo T. Saigusa D. Kato H. Suzuki K. (2020). Ferroptosis is controlled by the coordinated transcriptional regulation of glutathione and labile iron metabolism by the transcription factor BACH1. J. Biol. Chem. 295 (1), 69–82. 10.1074/jbc.RA119.009548 31740582 PMC6952604

[B76] Ou R. Lu S. Wang L. Wang Y. Lv M. Li T. (2022). Circular RNA circLMO1 suppresses cervical cancer growth and metastasis by triggering miR-4291/ACSL4-mediated ferroptosis. Front. Oncol. 12, 858598. 10.3389/fonc.2022.858598 35321435 PMC8936435

[B77] Pan C. Chen G. Zhao X. Xu X. Liu J. (2022). lncRNA BBOX1-AS1 silencing inhibits esophageal squamous cell cancer progression by promoting ferroptosis via miR-513a-3p/SLC7A11 axis. Eur. J. Pharmacol. 934, 175317. 10.1016/j.ejphar.2022.175317 36216119

[B78] Ping H. Jia X. Ke H. (2022). A novel ferroptosis-related lncRNAs signature predicts clinical prognosis and is associated with immune landscape in pancreatic cancer. Front. Genet. 13, 786689. 10.3389/fgene.2022.786689 35330729 PMC8940287

[B79] Porter N. A. Caldwell S. E. Mills K. A. (1995). Mechanisms of free radical oxidation of unsaturated lipids. Lipids 30 (4), 277–290. 10.1007/BF02536034 7609594

[B80] Qi W. Li Z. Xia L. Dai J. Zhang Q. Wu C. (2019). LncRNA GABPB1-AS1 and GABPB1 regulate oxidative stress during erastin-induced ferroptosis in HepG2 hepatocellular carcinoma cells. Sci. Rep. 9 (1), 16185. 10.1038/s41598-019-52837-8 31700067 PMC6838315

[B81] Qiu X. Shi Q. Zhang X. Shi X. Jiang H. Qin S. (2022). LncRNA a2m-AS1 promotes ferroptosis in pancreatic cancer via interacting with PCBP3. Mol. Cancer Res. 20 (11), 1636–1645. 10.1158/1541-7786.MCR-22-0024 35920801

[B82] Roh J. L. Kim E. H. Jang H. J. Park J. Y. Shin D. (2016). Induction of ferroptotic cell death for overcoming cisplatin resistance of head and neck cancer. Cancer Lett. 381 (1), 96–103. 10.1016/j.canlet.2016.07.035 27477897

[B83] Santana-Codina N. Gikandi A. Mancias J. D. (2021). The role of NCOA4-mediated ferritinophagy in ferroptosis. Adv. Exp. Med. Biol. 1301, 41–57. 10.1007/978-3-030-62026-4_4 34370287

[B84] Sato H. TaMba M. Ishii T. Bannai S. (1999). Cloning and expression of a plasma membrane cystine/glutamate exchange transporter composed of two distinct proteins. J. Biol. Chem. 274 (17), 11455–11458. 10.1074/jbc.274.17.11455 10206947

[B85] Sato K. Shi L. Ito F. Ohara Y. Motooka Y. Tanaka H. (2019). Non-thermal plasma specifically kills oral squamous cell carcinoma cells in a catalytic Fe(II)-dependent manner. J. Clin. Biochem. Nutr. 65 (1), 8–15. 10.3164/jcbn.18-91 31379408 PMC6667380

[B86] Shanshan W. Hongying M. Jingjing F. Yiming Y. Yu R. Rui Y. (2021). CircDTL functions as an oncogene and regulates both apoptosis and ferroptosis in non-small cell lung cancer cells. Front. Genet. 12, 743505. 10.3389/fgene.2021.743505 34621297 PMC8490767

[B87] Shen Z. Song J. Yung B. C. Zhou Z. Wu A. Chen X. (2018). Emerging strategies of cancer therapy based on ferroptosis. Adv. Mater 30 (12), e1704007. 10.1002/adma.201704007 29356212 PMC6377162

[B88] Song Z. Jia G. Ma P. Cang S. (2021). Exosomal miR-4443 promotes cisplatin resistance in non-small cell lung carcinoma by regulating FSP1 m6A modification-mediated ferroptosis. Life Sci. 276, 119399. 10.1016/j.lfs.2021.119399 33781830

[B89] Sun X. Ou Z. Chen R. Niu X. Chen D. Kang R. (2016). Activation of the p62-Keap1-NRF2 pathway protects against ferroptosis in hepatocellular carcinoma cells. Hepatology 63 (1), 173–184. 10.1002/hep.28251 26403645 PMC4688087

[B90] Sun Y. Berleth N. Wu W. Schlutermann D. Deitersen J. Stuhldreier F. (2021). Fin56-induced ferroptosis is supported by autophagy-mediated GPX4 degradation and functions synergistically with mTOR inhibition to kill bladder cancer cells. Cell Death Dis. 12 (11), 1028. 10.1038/s41419-021-04306-2 34716292 PMC8556316

[B91] Sung H. Ferlay J. Siegel R. L. Laversanne M. Soerjomataram I. Jemal A. (2021). Global cancer statistics 2020: GLOBOCAN estimates of incidence and mortality worldwide for 36 cancers in 185 countries. CA Cancer J. Clin. 71 (3), 209–249. 10.3322/caac.21660 33538338

[B92] Tan L. Mai D. Zhang B. Jiang X. Zhang J. Bai R. (2019). PIWI-interacting RNA-36712 restrains breast cancer progression and chemoresistance by interaction with SEPW1 pseudogene SEPW1P RNA. Mol. Cancer 18 (1), 9. 10.1186/s12943-019-0940-3 30636640 PMC6330501

[B93] Tan P. Li M. Liu Z. Zhao L. Fu W. (2022). Glycolysis-related linc02432/hsa-miR-98-5p/HK2 Axis inhibits ferroptosis and predicts immune infiltration, tumor mutation burden, and drug sensitivity in pancreatic adenocarcinoma. Front. Pharmacol. 13, 937413. 10.3389/fphar.2022.937413 35795552 PMC9251347

[B94] Tuo Q. Z. Lei P. Jackman K. A. Li X. L. Xiong H. Li X. L. (2017). Tau-mediated iron export prevents ferroptotic damage after ischemic stroke. Mol. Psychiatry 22 (11), 1520–1530. 10.1038/mp.2017.171 28886009

[B95] Venkataramani V. Yang Y. Schubert M. C. Reyhan E. Tetzlaff S. K. Wismann N. (2022). Glioblastoma hijacks neuronal mechanisms for brain invasion. Cell 185 (16), 2899–2917.e31. 10.1016/j.cell.2022.06.054 35914528

[B96] Wakabayashi N. Itoh K. Wakabayashi J. Motohashi H. Noda S. Takahashi S. (2003). Keap1-null mutation leads to postnatal lethality due to constitutive Nrf2 activation. Nat. Genet. 35 (3), 238–245. 10.1038/ng1248 14517554

[B97] Wang L. Wu S. He H. Ai K. Xu R. Zhang L. (2022). CircRNA-ST6GALNAC6 increases the sensitivity of bladder cancer cells to erastin-induced ferroptosis by regulating the HSPB1/P38 axis. Lab. Invest. 102, 1323–1334. 10.1038/s41374-022-00826-3 35945269

[B98] Wang Q. Chen J. Singh S. Xie Z. Qin F. Shi X. (2022). Profile of chimeric RNAs and TMPRSS2-ERG e2e4 isoform in neuroendocrine prostate cancer. Cell Biosci. 12 (1), 153. 10.1186/s13578-022-00893-5 36088396 PMC9463804

[B99] Wang Q. Li Z. Yang J. Peng S. Zhou Q. Yao K. (2021). Loss of NEIL3 activates radiotherapy resistance in the progression of prostate cancer. Cancer Biol. Med. 19 (8), 1193–1210. 10.20892/j.issn.2095-3941.2020.0550 34591415 PMC9425180

[B100] Wang W. Xu R. Zhao H. Xiong Y. He P. (2022). CircEXOC5 promotes ferroptosis by enhancing ACSL4 mRNA stability via binding to PTBP1 in sepsis-induced acute lung injury. Immunobiology 227 (4), 152219. 10.1016/j.imbio.2022.152219 35709678

[B101] Wang Y. Chen H. Wei X. (2021). Circ_0007142 downregulates miR-874-3p-mediated GDPD5 on colorectal cancer cells. Eur. J. Clin. Invest. 51 (7), e13541. 10.1111/eci.13541 33797091

[B102] Wang Y. Zhang S. Bai Y. Li G. Wang S. Chen J. (2022). Development and validation of ferroptosis-related LncRNA biomarker in bladder carcinoma. Front. Cell Dev. Biol. 10, 809747. 10.3389/fcell.2022.809747 35309945 PMC8924052

[B103] Wang Z. Chen X. Liu N. Shi Y. Liu Y. Ouyang L. (2021). A nuclear long non-coding RNA LINC00618 accelerates ferroptosis in a manner dependent upon apoptosis. Mol. Ther. 29 (1), 263–274. 10.1016/j.ymthe.2020.09.024 33002417 PMC7791008

[B104] Wei D. Ke Y. Q. Duan P. Zhou L. Wang C. Y. Cao P. (2021). MicroRNA-302a-3p induces ferroptosis of non-small cell lung cancer cells via targeting ferroportin. Free Radic. Res. 55 (7), 821–830. 10.1080/10715762.2021.1947503 34181495

[B105] Wu P. Li C. Ye D. M. Yu K. Li Y. Tang H. (2021). Circular RNA circEPSTI1 accelerates cervical cancer progression via miR-375/409-3P/515-5p-SLC7A11 axis. Aging (Albany NY) 13 (3), 4663–4673. 10.18632/aging.202518 33534779 PMC7906137

[B106] Wu Z. Lu Z. Li L. Ma M. Long F. Wu R. (2021). Identification and validation of ferroptosis-related LncRNA signatures as a novel prognostic model for colon cancer. Front. Immunol. 12, 783362. 10.3389/fimmu.2021.783362 35154072 PMC8826443

[B107] Xi Y. Shen Y. Wu D. Zhang J. Lin C. Wang L. (2022). CircBCAR3 accelerates esophageal cancer tumorigenesis and metastasis via sponging miR-27a-3p. Mol. Cancer 21 (1), 145. 10.1186/s12943-022-01615-8 35840974 PMC9284725

[B108] Xie Y. Zhu S. Song X. Sun X. Fan Y. Liu J. (2017). The tumor suppressor p53 limits ferroptosis by blocking DPP4 activity. Cell Rep. 20 (7), 1692–1704. 10.1016/j.celrep.2017.07.055 28813679

[B109] Xu P. Ge F. H. Li W. X. Xu Z. Wang X. L. Shen J. L. (2022). MicroRNA-147a targets SLC40A1 to induce ferroptosis in human glioblastoma. Anal. Cell Pathol. (Amst) 2022, 2843990. 10.1155/2022/2843990 35942174 PMC9356897

[B110] Xu P. Wang Y. Deng Z. Tan Z. Pei X. (2022). MicroRNA-15a promotes prostate cancer cell ferroptosis by inhibiting GPX4 expression. Oncol. Lett. 23 (2), 67. 10.3892/ol.2022.13186 35069876 PMC8756426

[B111] Xu Q. Zhou L. Yang G. Meng F. Wan Y. Wang L. (2020). CircIL4R facilitates the tumorigenesis and inhibits ferroptosis in hepatocellular carcinoma by regulating the miR-541-3p/GPX4 axis. Cell Biol. Int. 44 (11), 2344–2356. 10.1002/cbin.11444 32808701

[B112] Yadav P. Sharma P. Sundaram S. Venkatraman G. Bera A. K. Karunagaran D. (2021). SLC7A11/xCT is a target of miR-5096 and its restoration partially rescues miR-5096-mediated ferroptosis and anti-tumor effects in human breast cancer cells. Cancer Lett. 522, 211–224. 10.1016/j.canlet.2021.09.033 34571083

[B113] Yang H. Hu Y. Weng M. Liu X. Wan P. Hu Y. (2022). Hypoxia inducible lncRNA-CBSLR modulates ferroptosis through m6A-YTHDF2-dependent modulation of CBS in gastric cancer. J. Adv. Res. 37, 91–106. 10.1016/j.jare.2021.10.001 35499052 PMC9039740

[B114] Yang W. S. Kim K. J. Gaschler M. M. Patel M. Shchepinov M. S. Stockwell B. R. (2016). Peroxidation of polyunsaturated fatty acids by lipoxygenases drives ferroptosis. Proc. Natl. Acad. Sci. U. S. A. 113 (34), E4966–E4975. 10.1073/pnas.1603244113 27506793 PMC5003261

[B115] Yang W. S. SriRamaratnam R. Welsch M. E. Shimada K. Skouta R. Viswanathan V. S. (2014). Regulation of ferroptotic cancer cell death by GPX4. Cell 156 (1-2), 317–331. 10.1016/j.cell.2013.12.010 24439385 PMC4076414

[B116] Yang W. S. Stockwell B. R. (2016). Ferroptosis: Death by lipid peroxidation. Trends Cell Biol. 26 (3), 165–176. 10.1016/j.tcb.2015.10.014 26653790 PMC4764384

[B117] Yao J. Chen X. Liu X. Li R. Zhou X. Qu Y. (2021). Characterization of a ferroptosis and iron-metabolism related lncRNA signature in lung adenocarcinoma. Cancer Cell Int. 21 (1), 340. 10.1186/s12935-021-02027-2 34217273 PMC8254945

[B118] Yao W. Wang J. Meng F. Zhu Z. Jia X. Xu L. (2021). Circular RNA CircPVT1 inhibits 5-fluorouracil chemosensitivity by regulating ferroptosis through MiR-30a-5p/FZD3 Axis in esophageal cancer cells. Front. Oncol. 11, 780938. 10.3389/fonc.2021.780938 34966683 PMC8711269

[B119] Yu R. Zhou Y. Shi S. Wang X. Huang S. Ren Y. (2022). Icariside II induces ferroptosis in renal cell carcinoma cells by regulating the miR-324-3p/GPX4 axis. Phytomedicine 102, 154182. 10.1016/j.phymed.2022.154182 35636172

[B120] Zhang B. Bao W. Zhang S. Chen B. Zhou X. Zhao J. (2022). LncRNA HEPFAL accelerates ferroptosis in hepatocellular carcinoma by regulating SLC7A11 ubiquitination. Cell Death Dis. 13 (8), 734. 10.1038/s41419-022-05173-1 36008384 PMC9411508

[B121] Zhang D. L. Ghosh M. C. Rouault T. A. (2014). The physiological functions of iron regulatory proteins in iron homeostasis - an update. Front. Pharmacol. 5, 124. 10.3389/fphar.2014.00124 24982634 PMC4056636

[B122] Zhang H. Deng T. Liu R. Ning T. Yang H. Liu D. (2020). CAF secreted miR-522 suppresses ferroptosis and promotes acquired chemo-resistance in gastric cancer. Mol. Cancer 19 (1), 43. 10.1186/s12943-020-01168-8 32106859 PMC7045485

[B123] Zhang H. Ge Z. Wang Z. Gao Y. Wang Y. Qu X. (2021). Circular RNA RHOT1 promotes progression and inhibits ferroptosis via mir-106a-5p/STAT3 axis in breast cancer. Aging (Albany NY) 13 (6), 8115–8126. 10.18632/aging.202608 33686957 PMC8034942

[B124] Zhang K. Wu L. Zhang P. Luo M. Du J. Gao T. (2018). miR-9 regulates ferroptosis by targeting glutamic-oxaloacetic transaminase GOT1 in melanoma. Mol. Carcinog. 57 (11), 1566–1576. 10.1002/mc.22878 30035324

[B125] Zhang R. Pan T. Xiang Y. Zhang M. Xie H. Liang Z. (2022). Curcumenol triggered ferroptosis in lung cancer cells via lncRNA H19/miR-19b-3p/FTH1 axis. Bioact. Mater 13, 23–36. 10.1016/j.bioactmat.2021.11.013 35224289 PMC8843976

[B126] Zhang X. Wang L. Li H. Zhang L. Zheng X. Cheng W. (2020). Crosstalk between noncoding RNAs and ferroptosis: New dawn for overcoming cancer progression. Cell Death Dis. 11 (7), 580. 10.1038/s41419-020-02772-8 32709863 PMC7381619

[B127] Zhang Y. Guo S. Wang S. Li X. Hou D. Li H. (2021). LncRNA OIP5-AS1 inhibits ferroptosis in prostate cancer with long-term cadmium exposure through miR-128-3p/SLC7A11 signaling. Ecotoxicol. Environ. Saf. 220, 112376. 10.1016/j.ecoenv.2021.112376 34051661

[B128] Zhang Y. Luo M. Cui X. O'Connell D. Yang Y. (2022). Long noncoding RNA NEAT1 promotes ferroptosis by modulating the miR-362-3p/MIOX axis as a ceRNA. Cell Death Differ. 29 (9), 1850–1863. 10.1038/s41418-022-00970-9 35338333 PMC9433379

[B129] Zhang Y. Shi J. Liu X. Feng L. Gong Z. Koppula P. (2018). BAP1 links metabolic regulation of ferroptosis to tumour suppression. Nat. Cell Biol. 20 (10), 1181–1192. 10.1038/s41556-018-0178-0 30202049 PMC6170713

[B130] Zhang Y. Swanda R. V. Nie L. Liu X. Wang C. Lee H. (2021). mTORC1 couples cyst(e)ine availability with GPX4 protein synthesis and ferroptosis regulation. Nat. Commun. 12 (1), 1589. 10.1038/s41467-021-21841-w 33707434 PMC7952727

[B131] ZhangLncRNA N. T-U. C. R. Uc. Xu M. Wang Y. (2022). LncRNA T-UCR uc.339/miR-339/slc7a11 Axis regulates the metastasis of ferroptosis-induced lung adenocarcinoma. J. Cancer 13 (6), 1945–1957. 10.7150/jca.65017 35399708 PMC8990432

[B132] Zheng S. Hu L. Song Q. Shan Y. Yin G. Zhu H. (2021). miR-545 promotes colorectal cancer by inhibiting transferring in the non-normal ferroptosis signaling. Aging (Albany NY) 13 (24), 26137–26147. 10.18632/aging.203801 34954694 PMC8751587

[B133] Zhong Y. Tian F. Ma H. Wang H. Yang W. Liu Z. (2020). FTY720 induces ferroptosis and autophagy via PP2A/AMPK pathway in multiple myeloma cells. Life Sci. 260, 118077. 10.1016/j.lfs.2020.118077 32810509

[B134] Zhu C. Song Z. Chen Z. Lin T. Lin H. Xu Z. (2022). MicroRNA-4735-3p facilitates ferroptosis in clear cell renal cell carcinoma by targeting SLC40A1. Anal. Cell Pathol. (Amst) 2022, 4213401. 10.1155/2022/4213401 35646516 PMC9135554

